# Modeling the patient mix for risk-adjusted CUSUM charts

**DOI:** 10.1177/09622802211053205

**Published:** 2022-03-10

**Authors:** Philipp Wittenberg

**Affiliations:** 1 Department of Mathematics and Statistics, Helmut Schmidt University, Germany

**Keywords:** Average run length, Parsonnet score, probability distribution, quality control charts, statistical process control

## Abstract

The improvement of surgical quality and the corresponding early detection of its changes is of increasing importance. To this end, sequential monitoring procedures such as the risk-adjusted CUmulative SUM chart are frequently applied. The patient risk score population (patient mix), which considers the patients’ perioperative risk, is a core component for this type of quality control chart. Consequently, it is important to be able to adapt different shapes of patient mixes and determine their impact on the monitoring scheme. This article proposes a framework for modeling the patient mix by a discrete beta-binomial and a continuous beta distribution for risk-adjusted CUSUM charts. Since the model-based approach is not limited by data availability, *any* patient mix can be analyzed. We examine the effects on the control chart’s false alarm behavior for more than 100,000 different scenarios for a cardiac surgery data set. Our study finds a negative relationship between the average risk score and the number of false alarms. The results indicate that a changing patient mix has a considerable impact and, in some cases, almost doubles the number of expected false alarms.

## 1 Introduction

**Figure 1.**
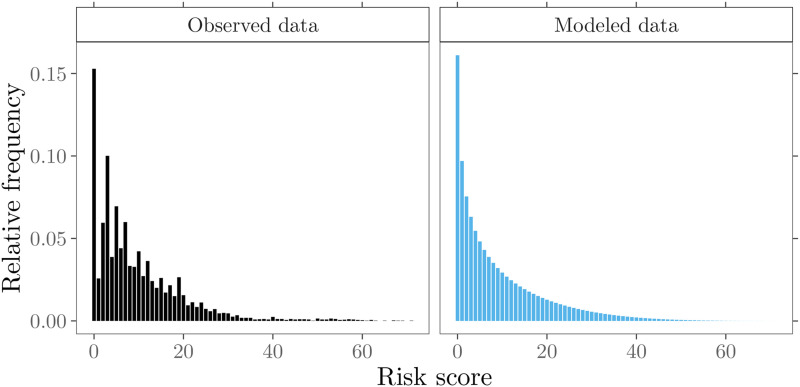
Cardiac surgery data. Relative frequencies of risk scores from the observed patient mix and modeled probabilities.

Improving the quality of health care, especially for surgical procedures, has become increasingly important. Monitoring methods from statistical process control, such as control charts, can support this task. One of the most widely used tools for this purpose is the risk-adjusted CUmulative SUM (RA CUSUM) chart developed by Steiner et al.^1^ It can quickly detect surgical performance changes by sequentially testing a log-likelihood ratio statistic adjusting for the perioperative risk of patients. In general, this risk is quantified by a risk scoring system where each patient is assigned an individual score. The resulting patient risk score population (patient mix) is subsequently used as a core component by the monitoring scheme. Since Steiner et al.^1^ presented the cardiac surgery data set (see the left panel in Figure 1), researchers have mainly used this particular patient mix in their studies. However, the data set has an inverse J-shape and represents only one empirical patient mix. This makes it difficult to consider and evaluate other scenarios. The paper’s main aim is to propose a flexible model-based approach for the patient mix to study the impact of changes on the number of false alarms of the monitoring scheme. Our modeling approach has high practical relevance as it overcomes the limitation to a single data set, allowing for investigations of different scenarios and more general conclusions.

The approach uses the beta-binomial and beta probability distribution to represent the patient mix on a discrete or continuous scale. Both the design and calibration of the RA CUSUM charts are simplified without losing their general insights. In addition to an identified or estimated risk model, knowledge of the patient risk distribution is required. For this purpose, it is much easier to set only a few model parameters of a valid probability distribution instead of collecting enough entries for all risk scores. Moreover, a modeling approach can reduce susceptibility to sampling errors due to artifacts or variability that may occur in the original data set, as shown in the left panel in Figure 1. Another motivation is the often encountered limited access to sensitive empirical data from the health care system due to data confidentiality. With our model-based approach, a patient mix can be transparently constructed by specifying the probability distribution and the following three parameters: maximum risk score and shape parameters α and β . Consequently, only this information needs to be provided to enable independent reproducibility of a patient mix.

**Figure 2.**
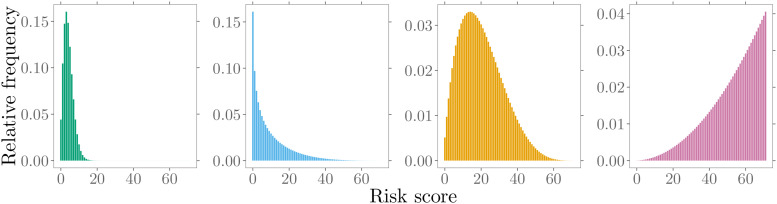
Different shapes of modeled patient risk score distributions.

Of broad interest are the impacts of individual RA components, i.e., the risk model or patient risk population, on the RA control charts’ characteristics. Inadequate choices or changes over time of these components can lead to increased number of false alarms. In practice, this often reduces the acceptance of the monitoring scheme. Previous studies, which took into account the variation in patient mix, examined only a small number of different scenarios. A key feature of our parametric modeling approach is the representation of various shapes of patient risk distributions by specific alterations of the model parameters, see Figure 2. In this way, a variety of patient risk distribution shapes can be accommodated, and unlike previous attempts, any number of scenarios can be evaluated.

We conduct a sensitivity analysis for the patient mix and gain insights into the false alarm behavior for more than 100,000 different scenarios for several control chart designs. Additionally, we examine the ability and speed of the monitoring scheme to detect various out-of-control situations. This study finds a negative relationship between the average risk score and the number of false alarms. The results indicate that a changing patient mix has a considerable impact, and in some cases, almost doubles the number of expected false alarms. In addition, we find that the proposed discrete and continuous distributions allow the patient mix to be modeled comparably well. It is shown that both choices have similar effects on the RA control chart performance.

The remaining part of this article is organized as follows. Section 2 gives an overview of the existing literature on modeling the patient mix and the heuristic generation of patient risk distributions. Section 3 provides background information on the data set used in our study and describes our modeling proposal. Section 4 introduces the RA CUSUM chart.

Accuracy comparisons for the numerical methods and patient mix models applied are given in Section 5. Section 6 presents a comprehensive sensitivity analysis of the patient mix. A simulation study in Section 7 demonstrates potential challenges in applying RA monitoring schemes. Section 8 contains some concluding remarks and discusses possible extensions. Further technical details can be found in Appendices A-D.

## 2 Related literature

In the context of statistical process monitoring, there are already excellent reviews of risk-adjusted procedures.^2–5^ Therefore, the following overview focuses on existing modeling approaches for the patient mix and the distributions that have been used. It also reviews earlier attempts to obtain different subgroups of patient populations from empirical data heuristically.

The use of probability distributions to model the patient mix for risk-adjusted (RA) control charts has had limited utilization in the past. Therefore, researchers mainly draw samples from the empirical distribution of the data. However, some probability distributions have been utilized. Hussein et al.^6^ assumed that the risk factor that characterizes the patient mix follows the Poisson distribution. Although this distribution is suitable for integer-based risk scores, its flexibility and fit to the data is often limited. Other researchers assumed a continuous risk score. While the application of the uniform^7,8^ and the normal^9,10^ distribution is of more theoretical than practical interest, the exponential distribution^11–13^ with its single parameter enables at least the representation of an inverse J-shaped patient mix. More advanced approaches consider a beta distribution.^14–16^ With a bounded interval and the ability to represent most of the shapes mentioned above, this distribution currently provides the most flexible approach for modeling a patient risk mix. However, integer risk scores that are approximated by assuming a continuous variable do not fully reflect the underlying data. In this context, we will present alternatives that directly consider the discrete nature of the risk scores.

Several researchers have indicated that a change in patient risk distribution, which is usually assumed to be constant over time, can affect the control chart’s performance to monitor surgical performance.^4,16–19,^ Previous studies, which took into account the variation in patient mix, often examined only a small number of different scenarios, which can be explained by limitations to simple manual manipulations or reclassifications of the used empirical data. Steiner et al. measured their RA approach’s sensitivity for the patient mix from two surgeons in their sample data with the most extreme patient mixes^1^ and for lowest risk and highest risk patients only.^20^ Chang^21^ divided the original data set into five risk categories and increased or decreased the number of patients within some categories by a fixed percentage to create new risk distributions of higher or lower risk. The author also obtained a series of risk distributions by combining a uniform or right-skewed distribution of patient numbers with different surgical risks. In their estimation error analysis for RA CUSUM, Jones and Steiner^22^ reduced the data set variability by examining only the lower end of the empirical risk distribution. They obtained the data for low-risk patients by restricting the sampling to risk scores ≤20 . To generate different patient mix populations, Tian et al.^18^ combined some of the previous approaches. The authors created five risk distributions, the first two years’ data, the lower 50% and the higher 50% of risk scores, and risk scores of the patients corresponding to the first and sixth surgeon. Further studies mainly adopted the procedures of Tian et al.^18^ and Jones and Steiner.^22^ By reversing the order of the sample data’s predisposed risk distribution, Rossi et al.^23^ obtained more patients at high-risk and fewer patients at low-risk. Knoth et al.^24^ created two new patient mixes by removing patients without other health conditions and all patients below the median risk score. Only Loke and Gan^16^ considered a continuous distribution to explicitly model the patient mix and examine possible effects for a small number of scenarios.

In this article, we extend and combine ideas from Loke and Gan^16^ and Tian et al.^18^ We propose a framework that overcomes the shortcomings mentioned earlier for patient mix modeling and provides a comprehensive analysis of the patient mix’s impact. The following section outlines our modeling approach, which considers the continuous beta distribution, a discretized beta, and the discrete beta-binomial distribution.

## 3 The modeling approach

### 3.1 Data

For illustration purposes of our modeling approach, we use the cardiac surgery operations data set presented in Steiner et al.^1^ It consists of records from 6994 patients in a single UK Hospital between 1992 and 1998 for seven operating surgeons. Available variables include survival time of each patient after an operation measured in days, the operating surgeon, date of operation, some risk factors such as age, diabetes, gender or number of reoperation, and the integer-based Parsonnet risk score.^25^ However, in our approach for modeling the patient risk mix, we only use the Parsonnet score, as it already contains the influential risk factors to characterize the individual risk of a patient. Alternatively, other risk assessment systems for cardiac surgery^4,26^ can also be combined with our modeling approach. As a measure of outcome, we consider the 30-day mortality. Accordingly, the binary operation outcome is expressed as y=0 for survival and y=1 for a patient’s death. Following an established procedure in statistical process monitoring, the data is split into two segments. The first 2 years 1992/93 ( 2218 operations) of the data (Phase I), are used to set up the RA control chart. The five-year 1994/98 (Phase II) monitoring period is often used to illustrate the actual monitoring of surgical performances. Note that one of the surgeon’s clinical records (#4) has only been available since mid-1997; and, it is not part of the Phase I data.

**Figure 3.**
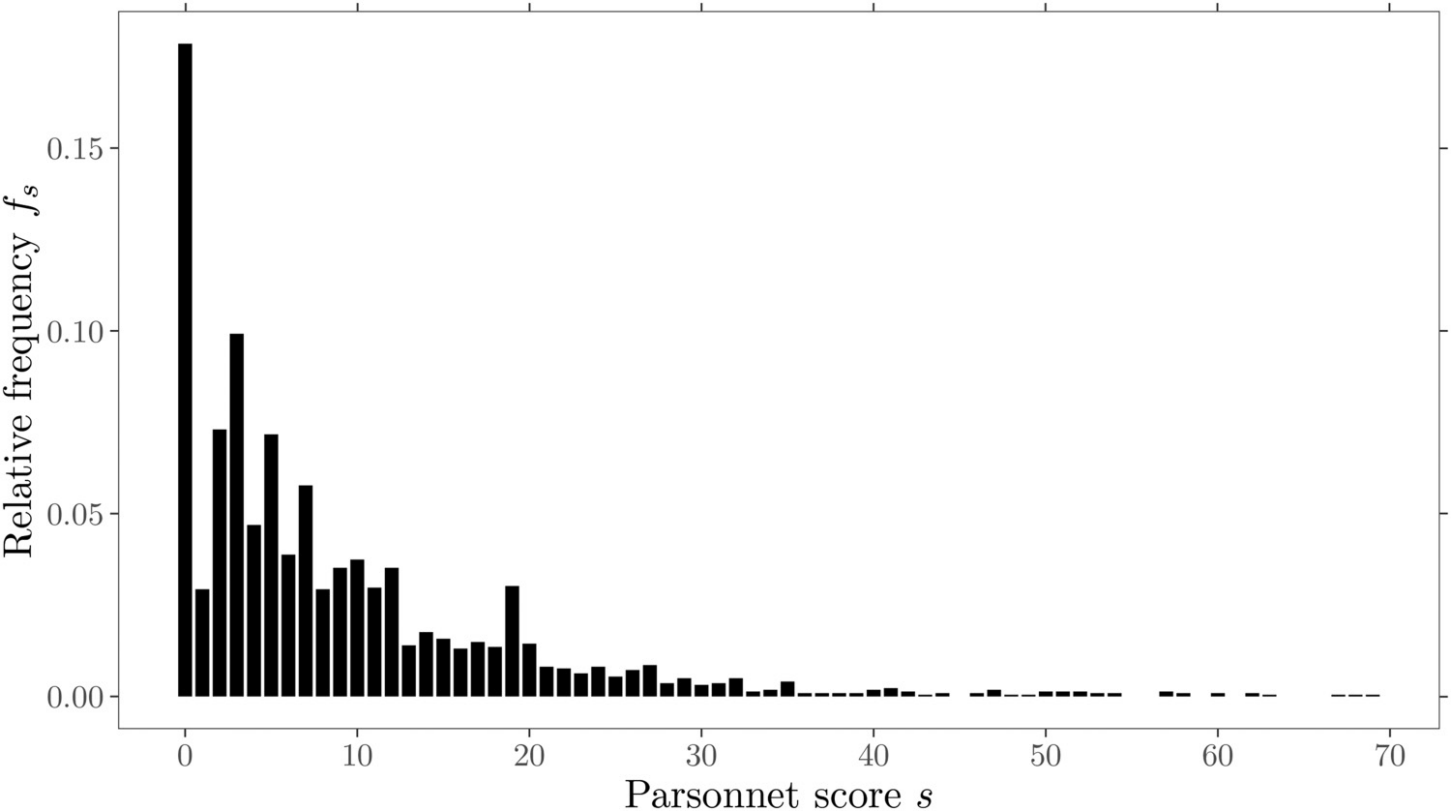
Cardiac surgery data set showing the relative frequencies of 2218 Parsonnet scores.

The relative frequencies $f_{s}$ of the Parsonnet score for the Phase I data are displayed in Figure 3. It is visible that the patient mix exhibits an inverse J-shape and is right-skewed. A total of 95% of the operations were carried out on patients with a risk score below 29 . It should be noted that there are some sparsely occupied risk scores in the remaining patients after s=45 . Furthermore, there are some prominent peak fluctuations visible (e.g. s=1,3,5,…) . A few of these peaks are likely related to the initial construction scheme and composition of the Parsonnet score. For example, a weight of three is added to the risk score for each of the risk factors: hypertension, morbid obesity, or diabetes. Also, factors like patient age or an unbalance of gender in the study population may cause some of these peaks. In summary, we can state that the patient mix shown in Figure 3 can be described by its 72 discrete risk scores ranging from 0 to 71 (full model). Note that Paynabar et al.^27^ argued that the Parsonnet risk scores in this data set do not follow any known probability distribution. We will elaborate in the next sections on how the patient mix can be modeled by either a continuous or a discrete parametric probability distribution.

### 3.2 Beta distribution

The univariate beta distribution,^28^ referred to as beta (α,β) , will be our initial choice to model the patient mix, since it was already used by some researchers. Furthermore, it has a bounded interval [0,1] for the random variable X and is flexible to adapt to different shapes of possible patient risk score populations. The probability density function (PDF) is given by

(1) $$f(x|\alpha ,\beta )=\frac{1}{B(\alpha ,\beta )}x^{\alpha -1}(1-x{)}^{\beta -1},\;0\leq x\leq 1,\alpha ,\beta >0$$

and the cumulative distribution function (CDF) is given by

(2) $$F(x,\alpha ,\beta )=\frac{B(x,\alpha ,\beta )}{B(\alpha ,\beta )}$$

where $B(\alpha ,\beta )=\int_{0}^{1}t^{\alpha -1}(1-t{)}^{\beta -1}dt$ is the complete beta function and the generalized form $B(x;\alpha ,\beta )=\int_{0}^{x}t^{\alpha -1}(1-t{)}^{\beta -1}dt$ is the incomplete beta function. The first two moments are defined as

(3) $$E(X)=\frac{\alpha }{\alpha +\beta },\;E(X^{2})=\frac{\alpha +1}{\left(\alpha +\beta +1\right)}\frac{\alpha }{\left(\alpha +\beta \right)}$$

To obtain parameter estimates for the beta distribution, a transformation of the integer risk scores into an interval (0,1] is required. In this work, the entire data’s maximum risk score, s=71 , is used to rescale s with $\frac{i+1/2}{71+1}i\in \{0,1,\ldots ,71\}$ to the interval midpoints. Then, the parameters α and β can be determined by the Method of Moments

(4) $$\hat{\alpha }=m_{1}(\frac{m_{1}(1-m_{1})}{m_{2}-m_{1}^{2}}-1),\;\hat{\beta }=(1-m_{1})(\frac{m_{1}(1-m_{1})}{m_{2}-m_{1}^{2}}-1)$$

with $m_{1}=1/K\sum_{i=1}^{K}X_{i}$ , $m_{2}=1/K\sum_{i=1}^{K}X_{i}^{2}$ , and the total number of patients K . By applying (4), we estimate $\hat{\alpha }=0.61$ and $\hat{\beta }=4.09$ . Furthermore, we propose the continuous beta distribution also for modeling in discrete form in order to account for the underlying original data structure. By dividing the beta distribution support [0,1] into 72 intervals of equal size and applying the CDF (2) with the estimated parameters $\hat{\alpha }=0.61$ and $\hat{\beta }=4.09$ , we obtain the related probabilities for the discrete risk scores.

### 3.3 Beta-binomial distribution

As a second probability distribution for modeling the patient mix, we propose the beta-binomial distribution introduced by Skellam.^29^ It has similar modeling flexibility as the beta distribution but uses discrete values that by design fit the Parsonnet integer risk score. It is a compound distribution where X∼ Bin (n,p) with n trials and p is a random variable with distribution beta (α,β) . The probability function is given by

$$\begin{array}{rl} P(x|n,\alpha ,\beta ) & =\left(\begin{array}{c} n\\ x \end{array}\right)\frac{1}{B(\alpha ,\beta )}\int_{0}^{1}\;p^{x+\alpha -1}{\left(1-p\right)}^{n-p+\beta -1}dp,\;\;\;\;n\in N,\;\alpha ,\beta >0\\ & =\left(\begin{array}{c} n\\ x \end{array}\right)\frac{B(\alpha +x,n+\beta -x)}{B(\alpha ,\beta )} \end{array}$$

The first two raw moments are given as

(5) $$E(X)=\frac{n\alpha }{\alpha +\beta },\;E(X^{2})=\frac{n\alpha (n(1+\alpha )+\beta )}{\left(\alpha +\beta )(\alpha +\beta +1\right)}$$

The point estimates for α and β can be obtained by the Method of Moments

(6) $$\hat{\alpha }=\frac{nm_{1}-m_{2}}{n(\frac{m_{2}}{m_{1}}-m_{1}-1)+m_{1}},\;\hat{\beta }=\frac{(n-m_{1})(n-\frac{m_{2}}{m_{1}})}{n(\frac{m_{2}}{m_{1}}-m_{1}-1)+m_{1}}$$

with $m_{1}=1/K\sum_{i=1}^{K}X_{i}$ , $m_{2}=1/K\sum_{i=1}^{K}X_{i}^{2}$ , and the total number of patients K . Note that $m_{1}$ , in this case, is the patient mix’s average risk score because, unlike the beta distribution, no rescaling of the original data is required. Comparable to the beta distribution, we set the maximum Parsonnet score s=n=71 . Accordingly, (6) leads to a parameterization of the beta-binomial distribution with $n=71,\hat{\alpha }=0.59$ , and $\hat{\beta }=4.12$ .

**Figure 4.**
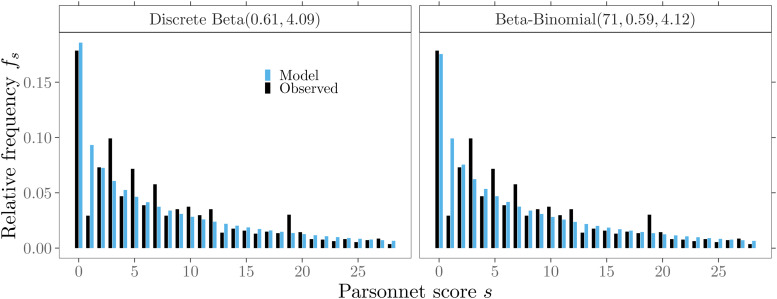
Probabilities of discrete patient mix models and observed relative frequencies for 95% of the Phase I data.

For a first evaluation of the model fit, we compare the modeled probabilities of the two discrete models: discrete beta( 0.61,4.09 ) and beta-binomial( 71,0.59,4.12 ) distribution with the relative frequencies of the original data. Figure 4 shows a graphical summary for 95% of the Phase I data ( s≤28 ). Despite the peak value fluctuations mentioned, both models fit quite well with the inverse J-shape, α<1 , of the empirical data (full model).

## 4 RA CUSUM and average run length calculation

The CUSUM scheme,^30^ a sequential procedure for rapid detection of changes, can be modified to consider specific risk factors.^31–34^ One of the most widely applied methods for this purpose is the RA Bernoulli CUSUM chart developed by Steiner et al.^1^ It is based on the log-likelihood ratio score and uses a risk model in which for patient i the risk score $s_{i}$ is used as an explanatory variable. The logistic regression model

(7) $$\mathrm{logit}\;\pi_{i}=\log\;\left(\frac{\pi_{i}}{1-\pi_{i}}\right)=b_{0}+b_{1}s_{i}$$

is usually applied. For the Phase I data, we obtain the regression coefficients (Maximum Likelihood) ${\hat{b}}_{0}=-3.6798$ and ${\hat{b}}_{1}=0.0768$ . Subsequently, the probability of death for a patient with Parsonnet score $s_{i}$ can be calculated by the inverse function

(8) $$\pi_{i}=(1+\exp\;(-b_{0}-b_{1}s_{i}){)}^{-1}$$

Following the approach of Steiner et al.,^1^ we compute the log-likelihood ratio statistic

$$W_{i}=\left\{ \begin{array}{cc} \log\;\left\{\frac{(1-\pi_{i}+Q_{0}\pi_{i})Q_{A}}{(1-\pi_{i}+Q_{A}\pi_{i})Q_{0}}\right\}, & \text{ if }\;y_{i}=1\\ \log\;\left\{\frac{1-\pi_{i}+Q_{0}\pi_{i}}{1-\pi_{i}+Q_{A}\pi_{i}}\right\}, & \text{ if }\;y_{i}=0 \end{array}\right.$$

using risk score $s_{i}$ in (8) and outcome $y_{i}$ and test the odds ratio under the null ( $Q_{0}$ ) and alternative Hypothesis ( $Q_{A}$ ). For $Q_{0}=1$ , W can be simplified to

(9) $$W_{i}=-\log\;(1-\pi_{i}+Q_{A}\pi_{i})+y_{i}\log\;Q_{A}$$

The CUSUM statistics $\{C_{i}^{+},C_{i}^{-}{\}}_{i=1,2,\ldots }$

(10) $$C_{i}^{+}=\max\{0,C_{i-1}^{+}+W_{i}^{+}\},\;C_{0}^{+}=0$$

(11) $$C_{i}^{-}=\min\{0,C_{i-1}^{-}-W_{i}^{-}\},\;C_{0}^{-}=0$$

give a signal when the upper or lower control limit h is exceeded. The upper-sided CUSUM chart signals, if $C_{i}^{+}>h^{+}$ and the lower-sided if, $C_{i}^{-}<-h^{-}$ . Each of the two CUSUM statistics (10), (11) can be designed and used separately to detect a specific shift in the odds ratio using $Q_{A}$ in (9), for example, to detect a deterioration ( $Q_{A}>1$ ) or an improvement ( $Q_{A}<1$ ) in surgical performance. However, usually, a two-sided design is used. The control limit is generally chosen based on a performance measure. In this study, we consider the Average Run Length (ARL). It is defined as the expected number of patients until a signal is given. When the surgical process is in-control, the ARL referred to as ${\mathrm{ARL}}_{0}$ should be high; otherwise, there would be too many false alarms. If an assignable cause is present and the process is out-of-control, the ARL referred to as ${\mathrm{ARL}}_{1}$ should be low to signal the change in the process quickly. In the following, we consider three different methods to approximate the ARL of RA CUSUM schemes.

The first method is the Monte Carlo simulation. It allows the determination of the control charts run-length under the assumptions of both a discrete and continuous distribution of the patient mix. However, especially for large ARL values, it requires a considerable amount of time to achieve a high precision level.^2,24^ Therefore, it is employed in this study only to verify the accuracy of the numerical methods used.

A second established method for calculating run-length properties is the Markov chain approach. It exploits the Markov property and can be applied to either a continuous or discrete random variable. The main idea is to discretize the CUSUM interval [0,h] by subintervals into a finite state space.^35^ The transition probabilities representing the possible changes within the control chart are combined into a transition matrix. The ARL can be computed by manipulating the matrix and solving a linear system of equations.^2^ The log-likelihood ratio score’s CDF derived in Appendix A is used to determine the continuous case’s transition probabilities. For the discrete case, a finite state space is generated by multiplying the irrational log-likelihood ratio scores (9) and the control limit h by a large number γ (scaling parameter), followed by appropriate rounding to the nearest integer. A reduction of the approximation error can be achieved by increasing the number of subintervals or the scaling parameter. However, this leads to an increased matrix dimension, which corresponds to increased computational complexity. Further technical details on the Markov chain approach can be found in Appendix C.

As a third method, applicable only to the continuous case, we utilize an integral equation approach to characterize the ARL . The basic idea is to consider a Fredholm integral equation of the second kind. It is solved numerically by the collocation method using Chebyshev polynomials of degree N . The degree of the polynomials determines the size of the linear system of equations. Thus, a larger N increases both the matrix dimension and the overall accuracy of the method. In some cases, the ARL function may not be smooth over the entire CUSUM continuation region, leading to considerable inaccuracies and instabilities in the ARL results. An extension, piece-wise collocation, can improve the approximation behavior by appropriately dividing the interval [0,h] into M subintervals before applying the actual collocation. However, since this procedure can increase the approximation’s stability and accuracy, it also features an enlarged matrix dimension NM of the linear system of equations. Further technical details on the collocation procedures can be found in Appendix B.

In the next section, the ARL approximation accuracy and stability of the individual methods, taking into account our modeling proposal’s distributions, are discussed and compared.

## 5 ARL approximation accuracy

Since our calculations are mainly based on numerical methods, we investigate the ARL approximation accuracy before further analysis.^2^ Webster and Pettitt^7^ have shown that factors such as degree of discretization of the CUSUM interval [0,h] , ARL size, and patient mix can affect the ARL’s approximation stability. Moreover, this investigation is conducted because the accuracy of results reported in the literature for the same setup varied widely.^24^

We follow Steiner et al.’s^1^ initial setup of the RA CUSUMs. These are two one-sided control charts, one to detect a shift in performance by doubling the odds ratio ( $Q_{A}=2$ ) and one by halving the odds ratio ( $Q_{A}=1/2$ ). It involves the Phase I data of the first 2 years, including 2218 operations, a risk model (7) with regression coefficients $b_{0}=-3.6798$ and $b_{1}=0.0768$ and control limits $h^{+}=4.5$ for the upper and $h^{-}=4$ for the lower RA CUSUM. The choice of these control limits leads to large in-control ARL values, which, due to the desired accuracy, result in large linear systems of equations expressed by large matrix dimensions and are therefore computationally intensive. However, numerical methods based on the Markov chain and the piece-wise collocation method can quickly compute the ARL. Incrementally increasing the resulting system dimension allows us to check the numerical approximations’ accuracy and stability for each setup: approximation method, patient mix model, and chart to detect deterioration or improvement. Additionally, all numerical methods are validated by Monte Carlo simulations with $10^{8}$ replications.

**Figure 5.**
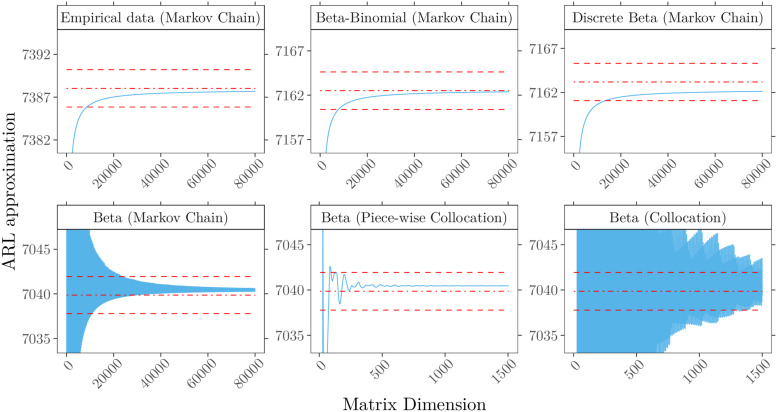
In-control ARL approximation for detecting deterioration ( $Q_{A}=2$ ) with control limit $h^{+}=4.5$ for different methods and patient mix models (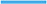). (Upper row) Discrete risk scores, computations by Markov chain approach for patient mix based on (top left) empirical data, (top) beta-binomial (71,0.59,4.12) and (top right) discrete beta (0.61,4.09) . (Lower row) Continuous risk scores modeled with beta (0.61,4.09) and approximated by (bottom left) Markov chain, (bottom) piece-wise collocation and (bottom right) full Collocation. Superimposed are Monte Carlo simulations with $10^{8}$ replications (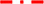) and three standard errors (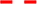).

**Figure 6.**
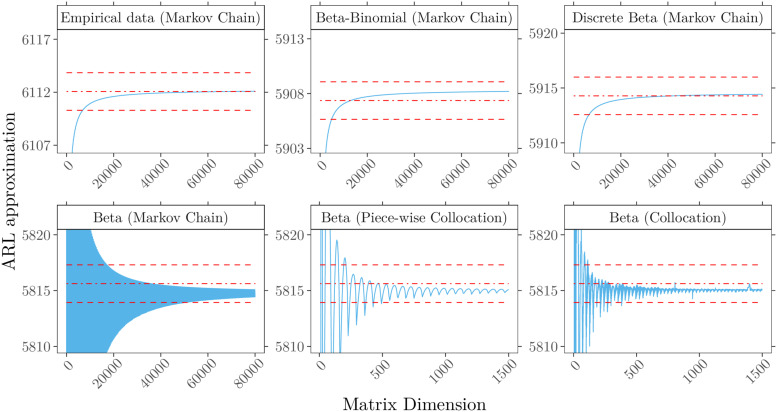
In-control ARL approximation for detecting improvement ( $Q_{A}=1/2$ ) with control limit $h^{-}=4$ for different methods and patient mix models (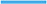). (Upper row) Discrete risk scores, computations by Markov chain approach for patient mix based on (top left) empirical data, (top) beta-binomial (71,0.59,4.12) and (top right) discrete beta( 0.61,4.09 ). (Lower row) Continuous risk scores modeled with beta (0.61,4.09) and approximated by (bottom left) Markov chain, (bottom) piece-wise collocation and (bottom right) full collocation. Superimposed are Monte Carlo simulations with $10^{8}$ replications (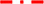) and three standard errors (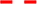).

Resulting ARL approximations for detecting deterioration are shown in Figure 5 and for improvement in Figure 6. The *x*-axis refers for each method to the size of the matrix dimensions used to solve the linear system of equations. It expresses the numerical effort required to achieve comparable accuracy and stability, regardless of the approximation method. In both Figures 5 and 6 (top left panel), we observe a divergence between the results reported by Steiner et al.^1^ and our ARL results for the full-score model. This is mainly related to the Markov chain’s matrix dimensions and rounding scheme. In Steiner et al., the ARL was computed using a simple rounding scheme and a Markov chain configuration with a matrix dimension of 250 . This results in an ARL equal to around 9600 for each chart. In this work, however, we employ a pairwise rounding scheme that provides more stable ARL approximations and is applied up to a matrix dimension of 80,000. The resulting ARL values are 7387.7 and 6112.1 , respectively. While an accuracy issue was first mentioned in Tian et al.,^18^ a detailed discussion is given in the Appendix in Knoth et al.^24^ Comparing the ARL approximation stability for $Q_{A}=2$ of the full model with discrete beta and beta-binomial in the upper row of Figure 5, we notice that all three methods behave similarly. They approach a slightly different level for matrix dimensions less than 20,000 relatively quickly. The lower row in Figure 5 compares approximation results for methods assuming a continuous patient mix modeled with the beta distribution. The piece-wise collocation method provides the most stable approximations. Polynomials of the degree N less than 100 leading to matrix dimensions less than 700 are sufficient to achieve a reasonable accuracy.

For the Markov chain method, a funnel-shaped approximation of the ARL is observed, with greater instability in the approximation behavior for smaller matrix dimensions. This suggests that a high degree of discretization of the CUSUM interval, corresponding to a large matrix dimension, is necessary to achieve the desired accuracy. The full collocation method does not approach a stable approximation, supporting the necessity of using one of these other two methods. For the detection of improvement, $Q_{A}=1/2$ , shown in Figure 6, all three discrete models (full-score, beta-binomial, and discrete beta) show a similarly smooth approximation behavior for the ARL as for $Q_{A}=2$ . Additional investigations for the numerical methods show that the beta-binomial distribution also provides stable ARL approximations for other model parameterizations. However, we observe an unstable approximation behavior for the beta distribution, especially for α<1 . This can be explained by the numerical integration challenges of the beta probability distribution. See Supplemental Material: Figures S.1 to S.6.

## 6 Sensitivity analysis of the patient mix

In this section, we first compare the full model with the model-based patient mixes ARL performance. The modeling approach is then applied to evaluate the patient mix’s sensitivity to risk distribution changes. In this way, we can determine the false alarm behavior of the monitoring scheme. Finally, we compare different patient risk distributions in their detection rates for various shifts in surgical performance.

### 6.1 Model comparison

The final ${\mathrm{ARL}}_{0}$ values for the approximation methods and patient mix models studied in the previous section are summarized in Table 1. It shows that the numerical methods’ final values are close to the Monte Carlo simulation results. The maximum standard error in simulation procedures is less than 0.71 . The constant gap in the ARL between the two CUSUM designs $Q_{A}=2$ and $Q_{A}=1/2$ is primarily related to the choice of specific control limits, $h^{+}=4.5$ and $h^{-}=4$ , from the illustrative example. To avoid this gap and enhance comparability, we calibrate all control charts in subsequent analyzes to an ${\mathrm{ARL}}_{0}$ of 7500 . Interestingly, we observe that the resulting ARL performance of RA CUSUM charts with a patient mix modeled by the proposed probability distributions deviates only between −3% and −5% from the full score model. For both models, which utilize either a discrete beta or beta-binomial distribution, the deviation from the full model is about −3% . The continuous beta distribution also exhibits only a small deviation of −5% . This result strongly supports our proposal to model the patient mix by a suitable probability distribution.

**Table 1.** Final in-control ARL values for patient distributions and approximation methods.

Patient distribution	Approximation method ${}^{*}$	${\mathrm{ARL}}_{0}$	
		$Q_{A}=2$	$Q_{A}=1/2$
Full model	MC-Simulation	7 388.0	6 112.1
	Markov chain	7387.7	6112.1
Beta-binomial (71,0.59,4.12)	MC-Simulation	7162.5	5907.4
	Markov chain	7162.4	5908.2
Discrete beta (0.61,4.09)	MC-Simulation	7163.2	5914.3
	Markov chain	7162.1	5914.4
Beta (0.61,4.09)	MC-Simulation	7039.9	5815.6
	Markov chain	7040.3	5814.6
	Piece-wise collocation	7040.5	5815.1
	Collocation	7039.4	5815.1

*MC-Simulation standard error <0.71 .

To investigate the effects of patient mixes on false alarm behavior and detection ability in the following sections, we choose the beta-binomial distribution, since it provides reliable and stable approximation results (see Figures 5, 6, and Table 1) as well takes into account the discrete data structure. However, almost comparable results for the following analysis can be obtained for a discrete beta distribution or a continuous beta distribution; see Supplemental Material (Figures S.7 to S.10 and Table T.1).

### 6.2 False alarm behavior

Several studies have indicated that a change in patient risk distribution, which is usually assumed to be constant, can affect the control chart’s performance to monitor surgical performance.^16–19^ To examine and quantify possible effects on the false alarm behavior ( ${\mathrm{ARL}}_{0}$ ), we perform a sensitivity analysis by modeling different patient mixes with a beta-binomial distribution.

A previous study^16^ used the beta (1,3) distribution to model the patient mix for a different data set. However, the analysis was restrictive in the number of scenarios examined ( α=1,β∈{2,2.5,4,5} ). To obtain greater insight into the ARL’s behavior concerning possible effects of changes in risk distribution, we take up the idea of Loke an Gan^16^ and investigate this problem on a larger scale. More specifically, we apply the Markov chain approximation method ( $\gamma =10^{4}$ ) for a beta-binomial (71,α,β) distribution. We set up a one-sided RA CUSUM chart tuned to detect a shift in the odds ratio of $Q_{A}=2$ and apply the risk model in (7) with $b_{0}=-3.6798$ and $b_{1}=0.0768$ . For the true model ( α=0.59,β=4.12 ) the target ${\mathrm{ARL}}_{0}$ is specified to 7500. An iterative procedure is applied based on a full grid search with four-digit decimal precision to determine the corresponding control limit $h^{+}=4.5443$ . Then, the ARLs of 102,771 different points (0.3≤α≤2,3≤β≤9) on a grid with step size 0.01 are calculated to determine their deviations from the target ${\mathrm{ARL}}_{0}$ .

**Figure 7.**
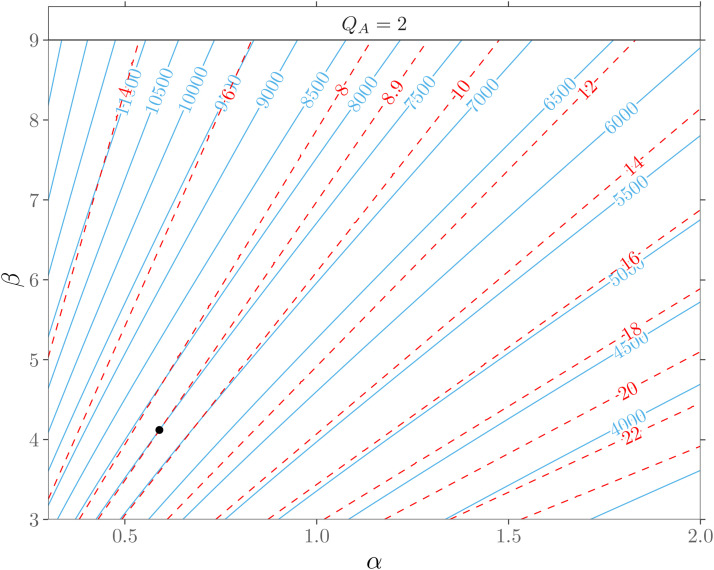
In-control ARLs for beta-binomial (71,α,β) models showing isolines of ARL (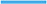) and expected risk score (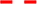). The control chart is calibrated for a beta-binomial (71,0.59,4.12) distribution ( ∙ ) to an ${\mathrm{ARL}}_{0}$ of 7500 .

Figure 7 shows the resulting isolines for the ${\mathrm{ARL}}_{0}$ and for the expected value (5) of the Parsonnet score following a beta-binomial (71,α,β) distribution. We observe that both types of isolines extend almost parallel as straight lines. Hence, the changes in the parameters describing the risk distribution may directly affect the ${\mathrm{ARL}}_{0}$ . These effects and their peculiarities can vary depending on the magnitude and direction of the parameter changes. For example, the in-control ARL reduces if either α increases and β=const or β decreases and α=const . This effect gets amplified when both parameters change in opposite directions ( α increase while β decrease). The three patterns of change described above correspond to a beta-binomial distribution, which models a risk distribution with an increased expected value. This corresponds to a patient population in which more operations are performed on average on patients with higher risk scores. Furthermore, simultaneous increases or decreases of α and β can have compensatory effects that only lead to relocation on the same ${\mathrm{ARL}}_{0}$ isoline. Thus the shape of the risk distribution has changed, but the expected value (5) remains constant, see Figure 7. In addition to these compensatory effects, more pronounced changes in the magnitude of a single shape parameter can also reduce or increase in the average risk score and, therefore, consequently lead to changes in the ${\mathrm{ARL}}_{0}$ . For example, the decrease of β may outweigh that of α , leading to an increase in the average risk score and reducing the in-control ARL. Inversion of the above-mentioned influences of the parameters α and β leads to contrary patterns of change in the risk distribution and an increased ${\mathrm{ARL}}_{0}$ . Hence, the control chart’s false alarm behavior is impacted less by the actual shape of risk distribution but primarily by the average risk score and its change.

Table 2 shows the impact of possible deviations from the first two years (Phase I) of the data set used as a calibrated reference scenario on the in-control ARL, similar to Figure 7. It further lists characteristics from different patient subsets of the original observed data and their related beta-binomial models. The upper part of the table includes risk distributions for six individual surgeons from the first two years of the data set and also for comparison, data from the remaining five years (Phase II), and the entire data set. Furthermore, we add two artificial risk mixes with extremely low and high-risk to illustrate the severity of possible effects.^18^ Except for one artificial patient mix, the inverse J-shaped form of the risk distribution ( α<1 ) remains unchanged in all subgroups. The patient mix characteristics (median and average risk score) from the original empirical and the beta-binomial model are also close. Substantial deviations from the ${\mathrm{ARL}}_{0}$ are not observed for Phase II and the entire data set, but for individual surgeons of Phase I in the range of −21% to +54% compared to the reference scenario. Deviations of the in-control ARL are similar for RA CUSUM control charts to detect deterioration or improvement, but slightly more pronounced in each subset for improvement, similar as in Tian et al.^18^ The lower part of Table 2 lists patient mixes of individual years from the year 1994 onwards to study changes of the risk distribution over time and validate potential effects. We observe that the annual patient mix is relatively constant and that only minor deviations ( −10% to +5% ) from the ${\mathrm{ARL}}_{0}=7500$ occur.

**Table 2.** Patient distribution characteristics (average and median risk score) and their corresponding in-control ARLs.

Patient distribution	Beta-binomial (71,α,β)			${\mathrm{ARL}}_{0}$		Empirical data		
	Parameter	Average	Median	$Q_{A}=2$	$Q_{A}=1/2$	Average	Median	Cases
Artificial high-risk	(1.50,4.00)	19.4	17	4342.0	3 983.0	−	−	−
Surgeon #2	(0.92,4.32)	12.5	10	6062.8	5902.2	12.4	10	287
Surgeon #1	(0.65,3.44)	11.3	8	6466.0	6255.3	11.3	7	565
Surgeon #7	(0.84,4.84)	10.5	7	6816.9	6793.6	10.5	8	260
Phase II	(0.77,4.83)	9.8	6	7134.8	7152.9	9.8	7	4776
Surgeon #3	(0.64,4.10)	9.6	6	7176.5	7130.9	9.6	6	324
Complete data	(0.71,4.59)	9.5	6	7235.7	7246.6	9.5	7	6994
Phase I*	(0.59,4.12)	**8.9**	**5**	**7500.5**	**7500.3**	**8.9**	**6**	**2218**
Surgeon #6	(0.58,6.87)	5.5	3	9731.5	10276.3	5.6	3	474
Surgeon #5	(0.53,8.14)	4.3	2	10759.2	11523.1	4.4	3	308
Artificial low-risk	(0.30,8.00)	2.6	1	12433.5	13483.3	−	−	−
Single year 1994	(0.68,3.90)	10.5	7	6761.9	6641.8	10.6	7	969
Single year 1995	(0.68,4.23)	9.8	7	7073.2	7027.8	9.9	7	1134
Single year 1996	(0.83,4.66)	10.7	7	6713.1	6661.4	10.7	8	877
Single year 1997	(0.97,6.35)	9.4	7	7382.6	7536.0	9.4	7	950
Single year 1998	(0.91,6.87)	8.3	6	7974.4	8241.0	8.3	6	846

*Reference scenario.

**Figure 8.**
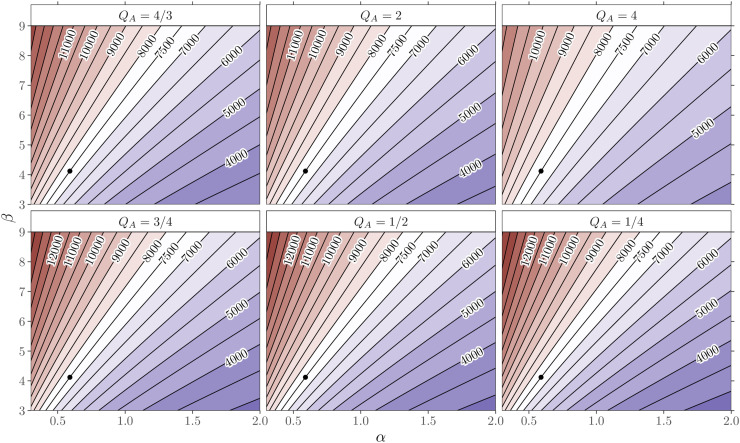
In-control ARLs for beta-binomial (71,α,β) models showing isolines of ARLs (—) for different out-of-control shift sizes $Q_{A}$ . All six charts are calibrated for a beta-binomial (71,0.59,4.12) distribution ( ∙ ) to an ${\mathrm{ARL}}_{0}$ of 7500 .

Next, we extend our investigations on the same α,β parameter scale as before and vary the CUSUM design parameter $Q_{A}$ , which controls the CUSUM chart’s sensitivity to detect a certain change in the odds ratio. Figure 8 shows the resulting ${\mathrm{ARL}}_{0}$ isolines for control charts designed to detect small, medium, and large shifts in surgical performance ( $Q_{A}=4/3,2,4$ ) with $h^{+}=2.9948,4.5443,5.7964$ and ( $Q_{A}=3/4,1/2,1/4$ ) with $h^{-}=2.8749,4.2252,5.1663$ , respectively. We note that the observed patterns of ${\mathrm{ARL}}_{0}$ isolines are similar to Figure 7 for all six charts independently of the considered shift size $Q_{A}$ . The choice of a CUSUM tuning parameter closer to one, $Q_{A}\approx Q_{0}$ , leads to larger deviations in the ${\mathrm{ARL}}_{0}$ (narrower isolines) when the patient mix has changed. Furthermore, the choice of a larger $Q_{A}$ leads to smaller deviations in the ARL profiles (wider isolines). This effect is somewhat less pronounced for detecting improvement $\left(Q_{A}<1\right)$ .

Based on our results, we confirm Tian et al.’s^18^ findings, namely that the $\mathrm{AR}L_{0}$ can vary considerably between different risk distributions and that there is a decreasing trend between $\mathrm{AR}L_{0}$ and average risk score. However, justified by our study’s scope, we can further generalize them. Moreover, by employing the isolines, we show a consistent (negative) relationship between the average risk score and $\mathrm{AR}L_{0}$ and that this relationship is valid independently of the design shift magnitude $Q_{A}$ .

### 6.3 Detection ability

Since the false alarm behavior of RA CUSUM charts can be significantly influenced by the patient mix, knowledge of the ability and speed of detection of changes in surgical performance, taking the risk distribution into account, is of valuable interest. Previous performance evaluations in the literature^20,21,23^ limited their examinations of out-of-control ARL ( ${\mathrm{ARL}}_{1}$ ) effects to a particular changed patient mix after online monitoring with the control chart had already started. In this scenario, regular recalibration of control limits is usually suggested to adapt to the new patient risk mix. However, comparisons of different patient risk distributions that may occur in different hospitals for the same type of surgery and assessing of their individual detection rate have not yet been performed.

Given the risk model in (7) with coefficients $b_{0}=-3.3798$ and $b_{1}=0.0768$ , we compare the performance of different patient populations modeled with the beta-binomial distribution. Again, Markov chain approximations ( $\gamma =10^{4}$ ) are used to calculate the ARLs of five different risk distributions. Three are derived from the original data, the complete Phase I, the two surgeons with the most extreme patient mix (Surgeon #2 and #5), and two artificial patient mixes with more extreme risk distributions, see Section 6.2. Various surgical performance levels $Q_{*}$ can be studied by modifying the odds ratio with

(12) $$\frac{\pi_{*}(s)}{1-\pi_{*}(s)}=Q_{*}\frac{\hat{\pi }(s)}{1-\hat{\pi }(s)}$$

where $Q_{*}<1$ characterizes improvement and $Q_{*}>1$ deterioration. In (12), the probability of death after surgery of a patient with a Parsonnet score s is given by $\hat{\pi }(s)$ (8) if performed by an average surgeon estimated from in-control data and $\pi_{*}(s)$ if performed by a surgeon with a changed surgical performance $Q_{*}$ . Subsequently, these modified probabilities are then used to calculate the log-likelihood ratio statistic in (9) and the CUSUM scores in (10), (11).

**Figure 9.**
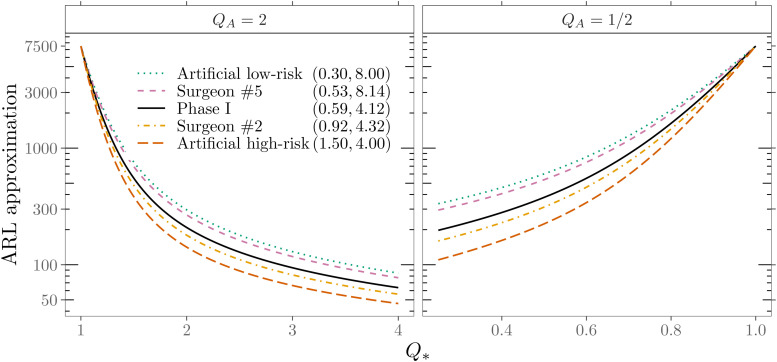
Out-of-control ARLs indicated on the logarithmic scale of one-sided RA CUSUM charts for five beta-binomial (71,α,β) models and different levels of surgical performance $Q_{*}$ . The left panel shows results for detecting deterioration and the right panel for detecting improvement in surgical performance.

Figure 9 depicts the resulting ARL profiles of one-sided RA CUSUM charts for $Q_{A}=2$ and $Q_{A}=1/2$ . For comparison, the in-control ARL of all charts is set to 7500 . Different levels of $Q_{*}$ are shown on a grid from 1/4 to 4 with stepsize 0.01 . We note that for very tiny changes ( $Q_{*}\approx Q_{0}$ ), there are only minor differences in the detection of changes in surgical performance between different patient mixes. More considerable changes in the odds ratio, characterized by a marked improvement or deterioration in surgical performance, are detected more quickly in RA CUSUM charts monitoring a higher risk patient mix. The spread of the ${\mathrm{ARL}}_{1}$ for specifc changes in the odds ratio $Q_{*}$ can be more than twice as high, depending on the patient mix’s risk distribution.

A summary of the setting $Q_{*}\in \{2,1/2\}$ is given in Table 3. From the ${\mathrm{ARL}}_{1}$ performance evaluation, we conclude that RA CUSUM control charts’ detection speed depends, similar to the ${\mathrm{ARL}}_{0}$ , mainly on the patient mix’s average risk score. Consequently, a change in surgical performance is more likely to be detected more quickly in a patient mix with a higher average risk score.

**Table 3.** Out-of-control ARL for different patient risk distributions.

Patient distribution	Beta-binomial (71,α,β)	$Q_{A},Q_{*}=2$		$Q_{A},Q_{*}=1/2$	
		$h^{+}$	${\mathrm{ARL}}_{1}$	$h^{-}$	${\mathrm{ARL}}_{1}$
Artificial low-risk	(0.30,8.00)	4.0636	296	3.6770	601
Surgeon #5	(0.53,8.14)	4.2001	267	3.8221	536	
Phase I	(0.59,4.12)	4.5443	209	4.2252	378
Surgeon #2	(0.92,4.32)	4.7494	179	4.4536	312	
Artificial high-risk	(1.50,4.00)	5.0736	142	4.8326	224

## 7 Simulation study

In this section, the model-based approach is utilized to develop alternative scenarios and highlight potential patient mix-related challenges when surgical performance is monitored online. For Phase I (training data), we assume a beta-binomial( 71,0.59,4.12 ) distribution to represent the patient mix and a risk model with coefficients $b_{0}=-3.3798$ and $b_{1}=0.0768$ . Two control charts $Q_{A}\in \{2,1/2\}$ with the control limits $h^{+}=4.5443$ and $h^{-}=4.2252$ are set up, giving an in-control ARL of 7500 each chart. Phase II (monitoring data) is generated by the model-based approach, and a change-point τ is introduced at patient number i=201 . This divides the data into two parts: in-control, i<τ , and out-of-control, i≥τ , where either a shift in the patient mix, surgical performance $Q_{*}$ , or both occurs:

$$\begin{array}{rl} \mathrm{for}\;i<\tau \;: & \;\;\;s\;∼\mathrm{beta}-\mathrm{binomial}(71,0.59,4.12),Q_{*}=1,\\ \mathrm{and}\;\mathrm{for}\;i\geq \tau \;: & \left\{ \begin{array}{cc} \;\;s\;∼\mathrm{beta}-\mathrm{binomial}(71,0.59,4.12),Q_{*}=2,\;(\mathrm{Scenario}\;A\;:\;\mathrm{const}.\;\mathrm{risk}\;\mathrm{mix}) & \\ s∼\mathrm{beta}-\mathrm{binomial}(71,1.50,4.00),Q_{*}=1,\;(\mathrm{Scenario}\;B\;:\;\mathrm{high}-\mathrm{risk}\;\mathrm{mix}) & \\ s∼\mathrm{beta}-\mathrm{binomial}(71,0.30,8.00),Q_{*}=2,\;(\mathrm{Scenario}\;C\;:\;\mathrm{low}-\mathrm{risk}\;\mathrm{mix}) & \end{array}\right. \end{array}$$

Two patient mixes of explicit lower-risk and higher-risk compared to the in-control patient mix were selected to demonstrate the effects of a single step change in risk distribution on the control charting procedure. However, we emphasize that the model-based approach allows investigations of any parameterization of patient mixes and shift patterns, i.e., multilevel or linear changes.

**Figure 10.**
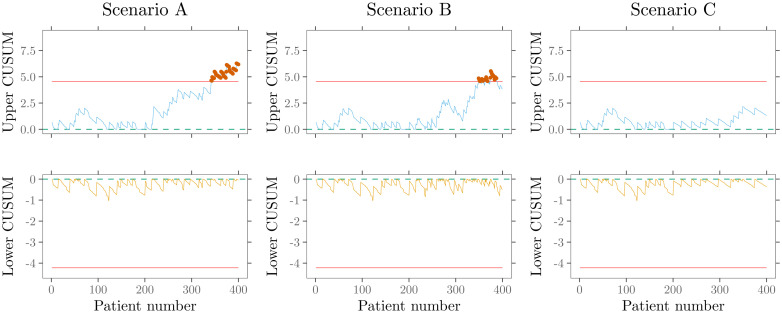
Online monitoring of surgical performance with RA CUSUM charts is displayed for three different scenarios. Scenario A (left panel) correctly signals a performance deterioration. Scenario B (middle panel) incorrectly signals performance deterioration. In scenario C (right panel), an actual change in performance is not detected.

Figure 10 shows the online monitoring of surgical performance in three different scenarios with RA CUSUM charts. The upper CUSUM charts are designed to detect deterioration, and the lower ones to detect an improvement in surgical performance. The left panel (*Scenario A*) displays the first signal at patient 342 , correctly indicating a deterioration in performance. In contrast, the middle panel (*Scenario B*) incorrectly signals performance deterioration at patient 349 , although the actual performance ( $Q_{*}=1$ ) has not changed, only the distribution to higher-risk patients. Furthermore, it is also possible to fully miss a change in performance. *Scenario C*’s control charts do not show a change in performance because the actual performance shift ( $Q_{*}=2$ ) overlays with a patient mix change to lower-risk patients. These examples clearly illustrate that it is challenging to distinguish whether the given signal is caused by an actual change in surgical performance or the patient mix. It also highlights the RA CUSUM charts’ fundamental problem namely its non-adaptability to risk distribution changes. Therefore, inferences from RA CUSUM control charts alone may be misleading or incorrect without considering the patient mix’s influence.^16^

## 8 Discussion

In this research, we propose a framework to flexibly model the patient risk score population using different probability distributions and investigate the effects of patient mix changes. Our study finds that both a beta-binomial and a discretized beta distribution are well suited to model the patient mix by the discrete risk scoring system’s underlying properties. However, a continuous beta-distributed risk score yields almost comparable results. In order to obtain reliable and precise results of the control chart performance measure ARL for the considered distributions, various numerical methods were applied and their approximation accuracy was checked. Because we employ a flexible parametric model instead of the complete empirical distribution, data availability is not an issue, and various patient mix changes can be analyzed. The sensitivity analysis of the patient mix with more than 100,000 different scenarios in Section 6 showed that the control chart’s false alarm behavior is less influenced by the actual shape of the risk distribution but primarily by the average risk score and its change. Specifically, the parallel isolines of in-control ARL and expected risk score exhibit a consistent negative relationship. This supports the application of a joint monitoring scheme of patient mix and surgical risk.^16^

We show that chart parameters, in this article, the control limit, based solely on Phase I patient mix data, should be used cautiously as Phase II data may differ from Phase I data. Accordingly, as shown in Section 6.2, the actual false alarm behavior can be severely affected. An alternative approach for setting up the control chart is to choose the desired ${\mathrm{ARL}}_{0}$ and then select the control limit that allows for such a false alarm rate. These Dynamic Probability Control Limits (DPCL) introduced by Zhang and Woodall^36^ in a RA setting can maintain the prespecified ${\mathrm{ARL}}_{0}$ throughout the whole monitoring period and are by design robust to patient mix changes. However, our proposed approach does not fix the issue of keeping the false alarm rate constant in the monitoring period. On the contrary, it highlights the classical’s RA Bernoulli CUSUM chart’s fundamental problem: the non-adaptability to changes in risk distribution from Phase I (training data) to Phase II (monitoring data). Thus, as shown in Section 7, conclusions from RA CUSUM charts alone that do not consider the patient mix’s influence or change can be misleading or incorrect. We recommend that users of RA CUSUM charts either apply DPCLs or a joint monitoring scheme of the surgical risk and the patient risk distribution.

The proposed modeling approach provides versatile opportunities for future research and extensions. On the one hand, it can be adapted for other CUSUM-type methods such as the RA CUSUM based on multiresponses^19,37^ or the E−O CUSUM.^38^ This also offers users of the variable life-adjusted display^33^ an application option. On the other hand, it can be a starting point for the development of new adaptive self-starting RA CUSUM charts that do not rely on Phase I data.

## Supplementary Material

Supplementary material

## Figures and Tables

**Figure 11.**
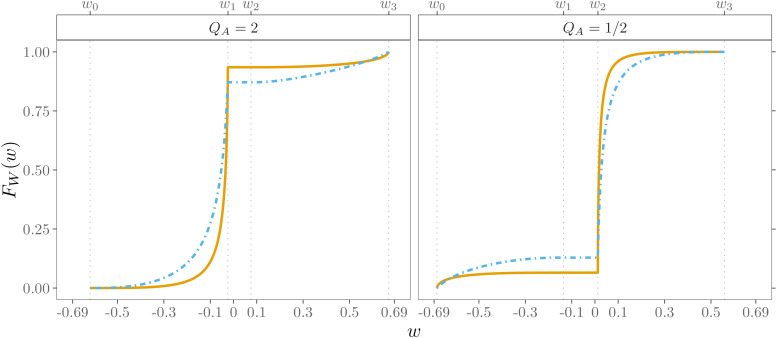
Cumulative distribution function $F_{W}(w)$ for beta (0.61,4.09) (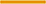) and beta (1,3) (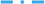) distribution, see (13), (14).

**Figure 12.**
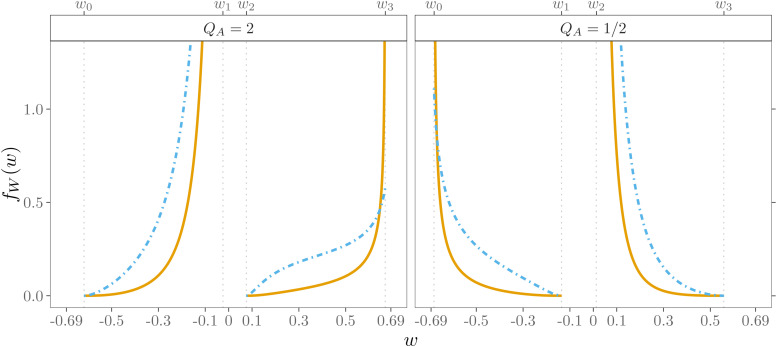
Probability density function $f_{W}(w)$ for beta (0.61,4.09) (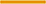) and beta (1,3) (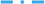) distribution, see (15), (16). Values for $f_{W}(w)$ are truncated at 1.3 .

**Figure 13.**
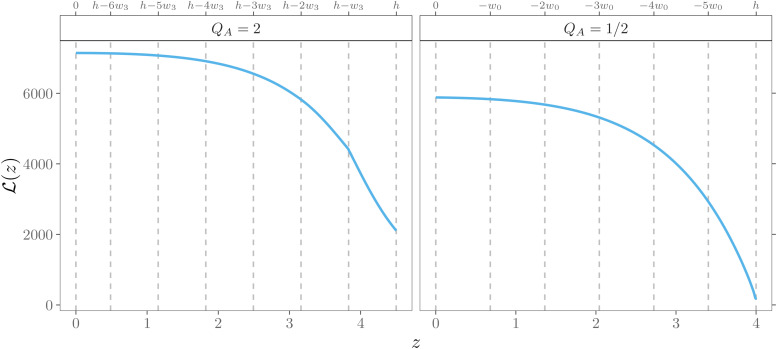
In-control ARL function L(z) (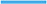) and piece-wise collocation intervals (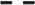).

## Appendices

### A Derivation of CDF and PDF for a continuous patient mix

In the following, we derive the cumulative distribution function $F_{W}(\cdot )$ for the log-likelihood ratio score W using a conditioning approach.^19^ This is exemplarily shown for $Q_{A}>1$ , but the CDF for detecting improvement can be derived in a similar way. The CUSUM increment (9) as a function of the (beta distributed) risk score s is written as follows

$$W(s)=\left\{ \begin{array}{cc} -\log\;(1+(Q_{A}-1)\pi (s)), & Y=0\\ -\log\;(1+(Q_{A}-1)\pi (s))+\log\;(Q_{A}), & Y=1 \end{array}\right.$$

As π(s)∈(0,1) in (8), hence for $Y=0,\pi \approx 1\;:\;-\log\;(Q_{A})$ and for $Y=1,\pi \approx 0\;:\;\log\;(Q_{A})$ , the theoretical range of W for $Q_{A}>1$ is $\left[-\log\;(Q_{A}),\log\;(Q_{A})\right]$ . However, 0<π(0),π(1)<1 reduces the possible range of W to

$$\begin{array}{rl} w_{0} & =-\log\;(1+(Q_{A}-1)\pi (1))\\ w_{1} & =-\log\;(1+(Q_{A}-1)\pi (0))\\ w_{2} & =w_{0}+\log\;(Q_{A})\\ w_{3} & =w_{1}+\log\;(Q_{A}) \end{array}$$

Hence, the support consists of two bounded intervals $\left[w_{0},w_{1}\right]$ , $\left[w_{2},w_{3}\right]$ . Define the inverse function(s) $W^{-1}(w)=:\;S(w)$

$$S(w)=\left\{ \begin{array}{cc} s_{0}(w)\;:=g(\frac{e^{-w}-1}{Q_{A}-1}), & Y=0,w\in [w_{0},w_{1}]\\ s_{1}(w)\;:=g(\frac{Q_{A}e^{-w}-1}{Q_{A}-1}), & Y=1,w\in [w_{2},w_{3}] \end{array}\right.$$

with

$$\begin{array}{rl} s_{0}(w_{0}) & =g(\frac{1+(Q_{A}-1)\pi (1)-1}{Q_{A}-1})=g(\pi (1))=1\\ s_{0}(w_{1}) & =g(\frac{1+(Q_{A}-1)\pi (0)-1}{Q_{A}-1})=g(\pi (0))=0 \end{array}$$

Recall that g is some suitably adjusted link function of the logit model

$$g(x)=-(b_{0}+\ln\;(x^{-1}-1))b_{1}^{-1}$$

Deploying total probability arguments to $F_{W}(\cdot )$ yields

$$\begin{array}{rl} F_{W}(w) & =P(W\leq w)=\int_{0}^{1}P(W\leq w|S=s)\cdot f_{S}(s)ds\\ & =\int_{0}^{1}(P(W\leq w|Y=0|S=s)\cdot \underset{=1-\pi (s)}{\underbrace{P(Y=0|S=s)}}\\ & \;\;+P(W\leq w|Y=1|S=s)\cdot \underset{=\pi (s)}{\underbrace{P(Y=1|S=s)}})\cdot f_{S}(s)ds \end{array}$$

We conclude that $P(W\leq w|Y=0|S=s)=1,\text{if}\;\text{w}\geq {\text{w}}_{1}$ and $P(W\leq w|Y=1|S=s)=0,\text{if}\;\text{w}\leq {\text{w}}_{2}$ . Furthermore, note that $s\geq s_{0}(w)\Leftrightarrow W(s)\leq w\;\text{for}\;\text{Y}=0,\text{w}\leq {\text{w}}_{1}$ and $s\geq s_{1}(w)\Leftrightarrow W(s)\leq w\;\text{for}\;\text{Y}=1,\text{w}\geq {\text{w}}_{2}$ .

For $w_{0}\leq w\leq w_{1}<w_{2}$ we conclude

$$F_{W}(w)=\int_{0}^{1}1\{s\geq s_{0}(w)\}\cdot (1-\pi (s))f_{S}(s)ds=\int_{s_{0}(w)}^{1}(1-\pi (s))f_{S}(s)ds$$

Analogously, for $w_{1}<w_{2}\leq w\leq w_{3}$ we derive

$$\begin{array}{rl} F_{W}(w) & =\int_{0}^{1}(1-\pi (s))f_{S}(s)ds+\int_{s_{1}(w)}^{1}\pi (s)f_{S}(s)ds\\ & =F_{W}(w_{1})+\int_{s_{1}(w)}^{1}\pi (s)f_{S}(s)ds \end{array}$$

Hence, the full CDF for $Q_{A}>1$ and $Q_{A}<1$ each split into two parts can be summarized as

$Q_{A}>1$ :

(13)
$$F_{W}(w)=\left\{ \begin{array}{cc} \int_{s_{0}(w)}^{1}(1-\pi (s))f_{S}(s)ds, & w_{0}\leq w\leq w_{1}\\ F_{W}(w_{1}), & w_{1}\leq w\leq w_{2}\\ F_{W}(w_{1})+\int_{s_{1}(w)}^{1}\pi (s)f_{S}(s)ds, & w_{2}\leq w\leq w_{3} \end{array}\right.$$

$Q_{A}<1$ :

(14)
$$F_{W}(w)=\left\{ \begin{array}{cc} \int_{0}^{s_{1}(w)}\pi (s)f_{S}(s)ds, & w_{0}\leq w\leq w_{1}\\ F_{W}(w_{1}), & w_{1}\leq w\leq w_{2}\\ F_{W}(w_{1})+\int_{0}^{s_{0}(w)}(1-\pi (s))f_{S}(s)ds, & w_{2}\leq w\leq w_{3} \end{array}\right.$$

Additional modifications in (13), (14) are required to achieve reasonable accuracy for a beta distribution with α<1 . The results were improved, in particular in the range near $w_{1}$ and $w_{3}$ , by applying a variable transformation, integration by parts, and a transformation of the argument of the integrand. Figure 11 illustrates $F_{W}(\cdot )$ for two parametrizations of the beta distribution for detecting deterioration (left panel) and improvement (right panel). By taking the first derivative of (13) and (14), the PDF $f_{W}(w)$ can be obtained for

$Q_{A}>1$ :

(15)
$$f_{W}(w)=\left\{ \begin{array}{ccc} -(1-\pi (s_{0}(w)))f_{S}(s_{0}(w))s_{0}^{\prime }(w), & & w_{0}\leq w\leq w_{1}\\ -\pi (s_{1}(w))f_{S}(s_{1}(w))s_{1}^{\prime }(w), & & w_{2}\leq w\leq w_{3}\\ 0, & & \text{elsewhere} \end{array}\right.$$

$Q_{A}<1$ :

(16)
$$f_{W}(w)=\left\{ \begin{array}{ccc} \pi (s_{1}(w))f_{S}(s_{1}(w))s_{1}^{\prime }(w), & & w_{0}\leq w\leq w_{1}\\ (1-\pi (s_{0}(w))f_{S}(s_{0}(w))s_{0}^{\prime }(w), & & w_{2}\leq w\leq w_{3}\\ 0, & & \text{elsewhere} \end{array}\right.$$

with $s_{0}^{\prime }(w)=\frac{\left(e^{-w}-1\right)}{b_{1}(Q_{A}-e^{-w})}\frac{e^{-w}}{\left(Q_{A}-1\right)}$ and $s_{1}^{\prime }(w)=\frac{\left(Q_{A}e^{-w}-1\right)}{b_{1}(Q_{A}-Q_{A}e^{-w})}\frac{Q_{A}e^{-w}}{\left(Q_{A}-1\right)}$ . Figure 12 illustrates $f_{W}(\cdot )$ for two parametrizations of the beta distribution for detecting deterioration (left panel) and improvement (left panel).

### B Collocation methods for a continuous patient mix

A procedure to approximate the ARL using an integral equation

$$L(u)=1+L(0)F_{W}(-u)+\int_{0}^{h}L(x)f_{W}(x-u)dx$$

was introduced by Page.^30^ In a risk-adjusted context,^15,16,19^ the collocation method^39^ can be applied to solve numerically the integral equation by the following linear system of equations

(17)
$$\sum_{\;j=1}^{N}c_{j}T_{j}^{*}(z_{i})=1+F_{W}(-z_{i})\sum_{\;j=1}^{N}c_{j}T_{j}^{*}(0)+\sum_{\;j=1}^{N}c_{j}\int_{0}^{h}T_{j}^{*}(x)f_{W}(x-z_{i})dx$$

The ARL function L(u) is approximated by $\sum_{\;j=1}^{N}c_{j}T_{j}^{*}(u),u\in [0,h]$ with Chebychev polynomials of degree N using $F_{W}$ and $f_{W}$ , where the first element of the vector L is the in-control ARL L(0) . As in Knoth^40^ we compute the Chebyshev nodes

$$z_{i}=\frac{h}{2}(1+\cos(\frac{(2-i)\pi }{2N}))$$

and Chebyshev polynomials

(18)
$$T_{j}(z)=\cos((j-1)\arccos(z)),\;z\in [-1;1]$$

which are transformed to the CUSUM continuation region (0,h)

$$T_{j}^{*}(z)=\cos((j-1)\;\;\arccos\;(\frac{2z-h}{h})),\;z\in [0;h]$$

An extension to the aforementioned collocation method is the piece-wise collocation method that has been successfully applied to approximate the ARL of CUSUM charts for $\chi^{2}$ distribution^40,41^ and Gamma distribution.^42^ Recently, Knoth^43^ showed its accuracy for a one-sided Exponential Weighted Moving Average chart and a beta distributed random variable. This improved accuracy is mainly achieved by a specific partitioning of the interval [0,h] into subintervals. For detecting a deterioration ( $Q_{A}>1$ ) we determine the number of intervals M and the interval borders, $\left[a_{i},b_{i}\right]$ i=1,…,M , based on the maximum increment of the CUSUM $w_{3}$ . Accordingly, we split up into $M=\lceil h/w_{3}\rceil$ subintervals with the interval borders $\left[h-(M-i-1)w_{3},h-(M-i)w_{3}\right]$ . This leads to M−1 equally spaced intervals from h backward and one shorter interval. For $Q_{A}<1$ we partition into $M=\lceil h/-w_{0}\rceil$ intervals with interval borders $\left[-w_{0}(i-1),-w_{0}i\right]$ . This generates M−1 equally spaced intervals from zero onwards and one shorter interval. Figure 13 shows the resulting 7 and 6 subintervals and ARL functions for $Q_{A}=2$ and $Q_{A}=1/2$ , respectively, for applying the piece-wise collocation method in Section 5.

The Chebyshev nodes are now computed in the i th subinterval $[a_{i},b_{i}],i=1,2,\ldots ,M$ by

$$z_{ij}=a_{i}+\frac{b_{i}-a_{i}}{2}[1+\cos(\frac{(M-1-j)\pi }{M-1})]$$

The Chebyshev polynomials in (18) are transformed and modified for each subinterval by

$$T_{ij}^{*}(z)=T_{j}(\frac{2z-a_{i}-b_{i}}{b_{i}-a_{i}}),\;z\in [a_{i};b_{i}],\;i=1,2,\ldots ,M$$

The resulting system of linear equations to determine the unknown constants $c_{ij}$ for detecting deterioration is given by

(19)
$$\begin{array}{rl} & \sum_{\;j^{\prime }=1}^{N}c_{ij^{\prime }}T_{ij^{\prime }}^{*}(z_{ij})=1+F_{W}(-z_{ij})\sum_{\;j=1}^{N}c_{1j^{\prime }}T_{1j^{\prime }}^{*}(0)\;+\sum_{i^{\prime }=\max(1,i-1)}^{\min(M,i+1)}\sum_{\;j^{\prime }=1}^{N}c_{i^{\prime }j^{\prime }}\int_{a_{i^{\prime }}}^{b_{i^{\prime }}}T_{i^{\prime }j^{\prime }}^{*}(x)f_{W}(x-z_{ij})dx \end{array}$$

with $a_{i^{\prime }}=\max\{a_{i^{\prime }},z_{ij}+w_{0}\}$ and $b_{i^{\prime }}=\min\{b_{i^{\prime }},z_{ij}+w_{3}\}$ . The in-control ARL $L_{0}$ can then be approximated by $\sum_{j=1}^{N}c_{1j}T_{j}^{*}(0)$ . As for the CDF $F_{W}$ in (13), (14), the integrals in (17), (19) are numerically approximated by Gauss Kronrod quadratures. However, given the divided and bounded density $f_{W}$ shown in Figure 12, further adjustments were made in (17) and (19) to improve the ARL approximation stability. This was particulary necessary for the beta distribution parameter α<1 , which was estimated for this data set in Section 3. The integral was first decomposed into two separate segments to omit the inner region $z_{ij}+w_{1}$ to $z_{ij}+w_{2}$ . Furthermore, quadratic substitutions were applied, which sufficiently stabilized the behavior of the integrand. For $Q_{A}>1$ we transformed $x=w_{1}-y^{2}$ in case of $f_{W}$ on $\left[w_{0},w_{1}\right]$ and $x=w_{3}-y^{2}$ in case of $f_{W}$ on $\left[w_{2},w_{3}\right]$ . Similar modifications were considered to detect improvement ( $Q_{A}<1)$ , $w_{0}$ , and $w_{2}$ as substitutions for the integrand's arguments and corresponding adjustments of the integral limits $a_{i}$ and $b_{i}$

### C Markov chain approximation

In the case of a discrete risk score distribution, such as in the full score, beta-binomial, and discrete beta model, the two CUSUM increments W in (9)

$$w_{s}^{\left(0\right)}=-\log\;(1+(Q_{A}-1)\pi (s)),\;w_{s}^{\left(1\right)}=w_{s}^{\left(0\right)}+\log\;Q_{A},$$

have unequal ranges. For this reason, we adopt the recently improved implementation of the Markov chain approximation of Knoth et al.,^24^ which is based on the scaling and rounding approach of Steiner et al.^1^ For $Q_{A}>1$ , all terms $w_{s}^{\left(0\right)},w_{s}^{\left(1\right)}$ for s∈{0,1,2,…,71} are ordered to

$$-\log\;Q_{A}<w_{71}^{\left(0\right)}<w_{70}^{\left(0\right)}<\ldots <w_{0}^{\left(0\right)}<0<w_{71}^{\left(1\right)}<w_{70}^{\left(1\right)}<\ldots <w_{0}^{\left(1\right)}<\log\;Q_{A}$$

Next, the CUSUM continuation region [0,h] is inflated by scaling with a large number γ , and the resulting $\gamma w_{s}^{\left(d\right)},d=0,1$ are rounded to the nearest integer value $\kappa_{s}^{\left(d\right)}$ . Thus the approximation sequence is set to a grid {0,1,…,t} , where t=γh is the absorbing state. The transition probability matrix

$$P=\left[\begin{array}{cc} Q & (I-Q)1\\ 0^{⊤} & 1 \end{array}\right]$$

can be created, where I=identitymatrixofdimensiont×t and 1,0 are column vectors of ones and zeros of dimension t . For the transition from i to j > 0, the submatrix

$$Q=\begin{array}{ccccc} & 0 & 1 & \cdots & t-1\\ 0 & (q_{00} & q_{01} & \cdots & q_{0,t-1}\\ 1 & q_{10} & q_{11} & \ddots & q_{1,t-1}\\ \vdots & \vdots & \vdots & \ddots & \vdots \\ t-1 & q_{t-1,0} & q_{t-1,1} & \cdots & q_{t-1,t-1}) \end{array},$$

is filled by checking $j-i\in \{\kappa_{71}^{\left(0\right)},\ldots ,\kappa_{0}^{\left(0\right)},\kappa_{71}^{\left(1\right)},\ldots ,\kappa_{0}^{\left(1\right)}\}=:\;\kappa$ . If this condition is true

(20)
$$q_{ij}=\left\{ \begin{array}{cc} \pi (s)f_{s}, & j>i\;(d=1)\\ (1-\pi (s))f_{s}, & j<i\;(d=0) \end{array}\right.$$

otherwise $q_{ij}=0$ , taking into account that several matching integer candidates are summed. The first column of Q is filled by

(21)
$$q_{i0}=\sum_{s\;:\;\kappa_{s}^{\left(0\right)}\leq -i}(1-\pi (s))f_{s}$$

The $f_{s}$ can be obtained either from the relative frequencies of the full model or from the probabilities from the PMF of the beta-binomial or discrete beta distribution. For $Q_{A}<1$ , the described procedure for filling the matrix Q can be applied analogously. Since in particular a paired rounding scheme^7,24^ leads to an improved ARL approximation accuracy for the RA CUSUM, we apply

$$\begin{array}{rl} {\underline{\kappa }}_{s}^{\left(d\right)} & \leq \gamma w_{s}^{\left(d\right)}<{\underline{\kappa }}_{s}^{\left(d\right)}+1=:\;{\bar{\kappa }}_{s}^{\left(d\right)},\;{\underline{\kappa }}_{s}^{\left(d\right)},{\bar{\kappa }}_{s}^{\left(d\right)}\in Z\\ f_{s} & =({\bar{\kappa }}_{s}^{\left(d\right)}-\gamma w_{s}^{\left(d\right)})f_{s}+(\gamma w_{s}^{\left(d\right)}-{\underline{\kappa }}_{s}^{\left(d\right)})f_{s}={\underline{f}}_{s}+{\bar{\;f}}_{s} \end{array}$$

and obtain an increased set κ for (20) and (21). In this rounding design, the absorbing state is determined by t=⌊γh⌋ . We follow Knoth et al.^24^ and add the resulting “excess” probability $p_{e}$ :=γh−t∈[0,1) , if present, into the last column of Q with $p_{e}\pi_{s}{\bar{f}}_{s}$ (or $p_{e}\pi_{s}{\underline{f}}_{s}$ ) to $q_{i,t-1}$ if there is a ${\bar{\kappa }}_{s}^{\left(d\right)}=t-i$ (or ${\underline{\kappa }}_{s}^{\left(d\right)}=t-i$ ). Finally, by solving a system of linear equations

(22)
$$L={\left(I-Q\right)}^{-1}1,$$

the in-control ARL $L_{0}$ , the first element of vector L , can be determined.^44^

For a continuous risk score distribution, the matrix Q is filled in the traditional way, namely

$$q_{ij}=\left\{ \begin{array}{cc} F_{W}(-i\omega +\omega /2), & i=0,\ldots ,t-1,j=0\\ F_{W}((j-i)\omega +\omega /2)-F_{W}((j-i)\omega -\omega /2), & i,j=1,\ldots ,t-1 \end{array}\right.$$

with matrix dimension t , interval size $\omega =\frac{2h}{2t-1}$ and the CDF $F_{W}(\cdot )$ of CUSUM increment (13), (14). The ARL can then be computed by equation (22).

Note that in addition to (22), the ARL can be computed by Markov chain approximations for both discrete and continuous risk score distributions using the algorithm recently published by Knoth et al.^24^ This recursive algorithm enables faster and more efficient computation of the ARL for large matrix dimensions utilizing the Toeplitz-like property of Q and the relationship between CUSUM and the sequential probability ratio test. This was favored in this article because of the large dimensions of the transition probability matrices.

### D Software

All computations of the presented results are carried out using R.^45^ The R-package vlad^46^ available in the Comprehensive R Archive Network (CRAN) contains all functions for calculating the ARL and the control limit h for the probability distributions and methods discussed. Computationally intensive algorithms were implemented with Rcpp^47^ and RcppArmadillo.^48^ The numerical approximations of integrals are performed using Gauss Kronrod quadratures implemented in the boost library accessed via the R-package BH.^49^ Graphics shown in the manuscript are created using ggplot2^50^ and metR^51^ for the isoline plots.
