# Supplementary material for: Modeling the patient mix for risk-adjusted CUSUM charts
Source: Stat Methods Med Res. 2022 Mar 10;31(5):779–800. doi: 10.1177/09622802211053205 (PMC9014690; doi:10.1177/09622802211053205)
Supplement: Supplementary material [file sj-pdf-1-smm-10.1177_09622802211053205.pdf]

Supplemental Material

Modeling the patient mix for risk-adjusted CUSUM charts

Philipp Wittenberg
Department of Mathematics and Statistics
Helmut Schmidt University
22043 Hamburg Germany
pwitten@hsu-hh.de

March 7, 2021

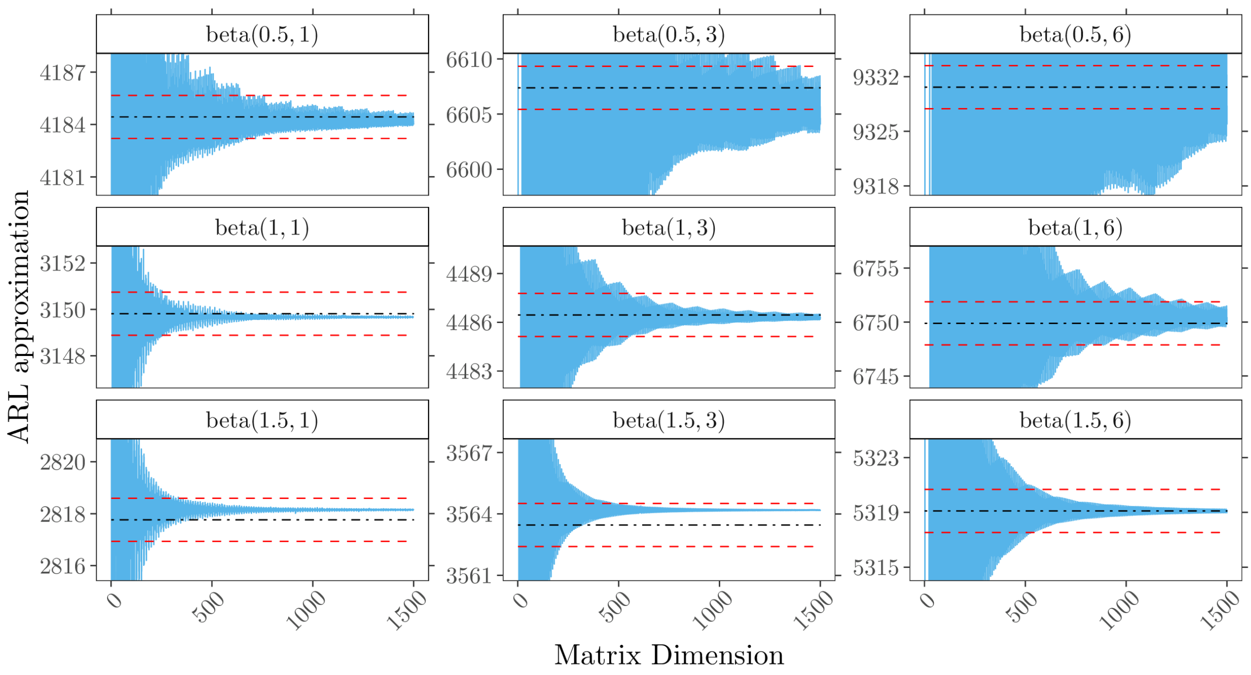

S. 1: In-control ARL approximation by collocation method (—) for detecting deterioration ($Q_A = 2$) and different beta(α, β) distributions with control limit $h^+ = 4.5$. Superimposed are Monte Carlo simulations with 10^8 replications (-·-) and three standard errors (- -).

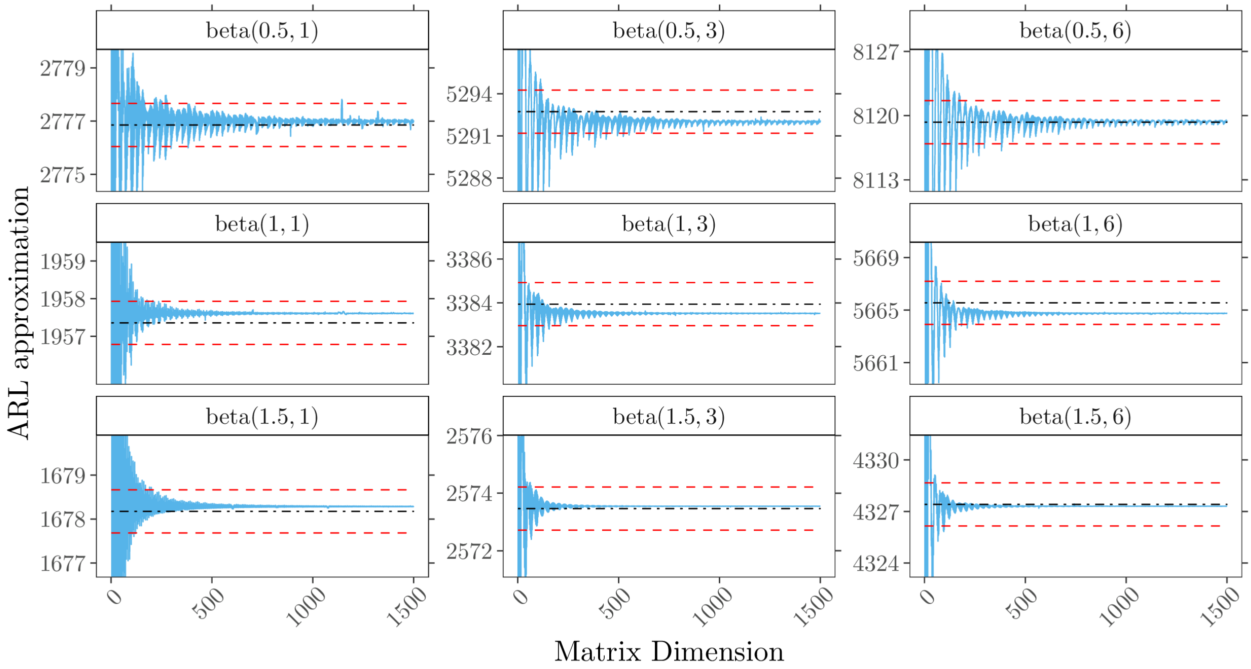

S. 2: In-control ARL approximation by collocation method (—) for detecting improvement ($Q_A = 1/2$) and different beta(α, β) distributions with control limit $h^- = 4$. Superimposed are Monte Carlo simulations with 10^8 replications (-·-) and three standard errors (- -).

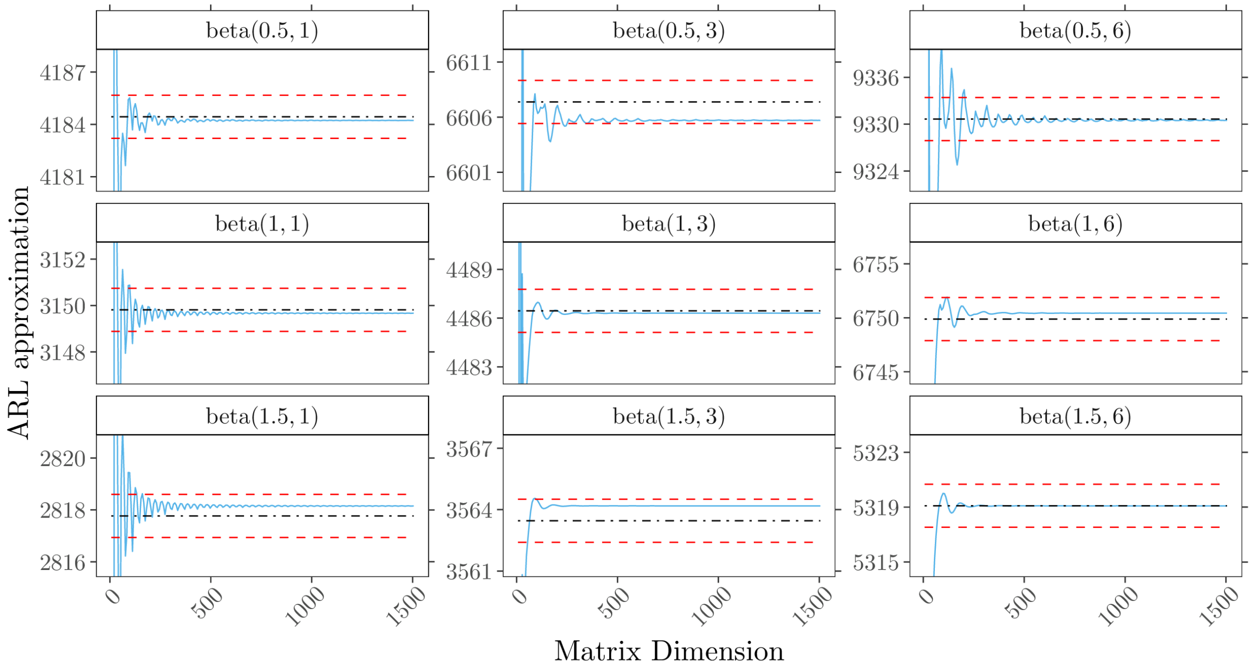

S. 3: In-control ARL approximation by piece-wise collocation method (—) for detecting deterioration ($Q_A = 2$) and different beta(α, β) distributions with control limit $h^+ = 4.5$. Superimposed are Monte Carlo simulations with 10^8 replications (-·-) and three standard errors (- -).

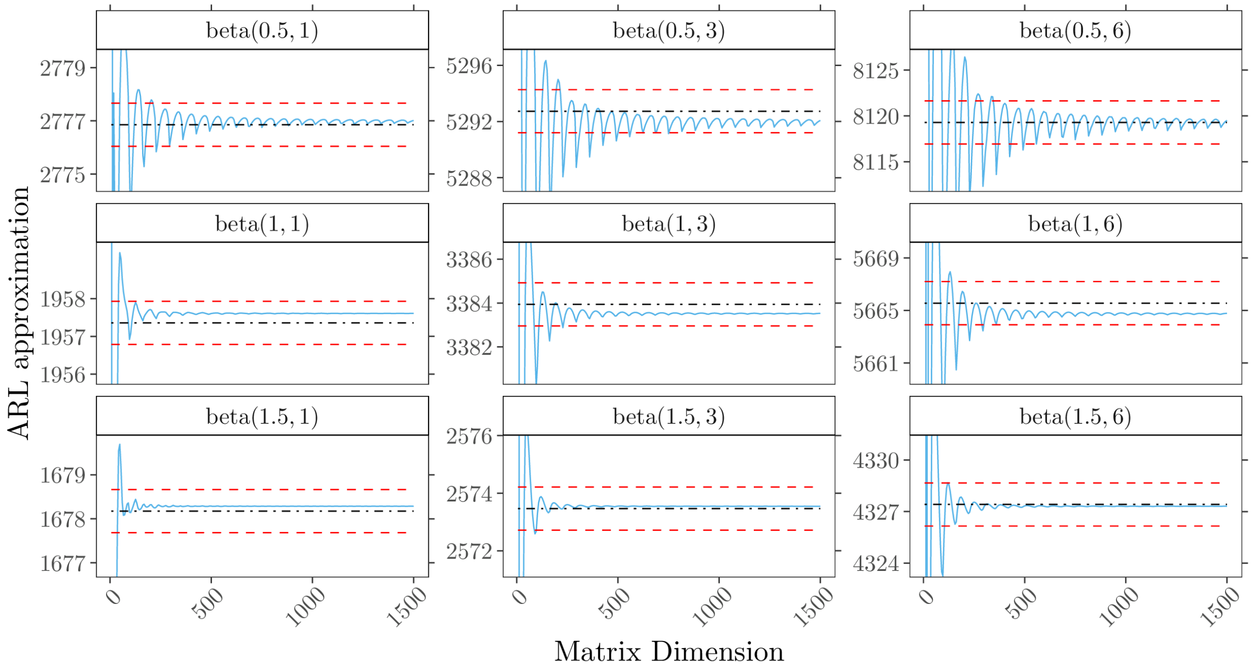

S. 4: In-control ARL approximation by piece-wise collocation method (—) for detecting improvement ($Q_A = 1/2$) and different beta(α, β) distributions with control limit $h^- = 4$. Superimposed are Monte Carlo simulations with 10^8 replications (-·-) and three standard errors (- -).

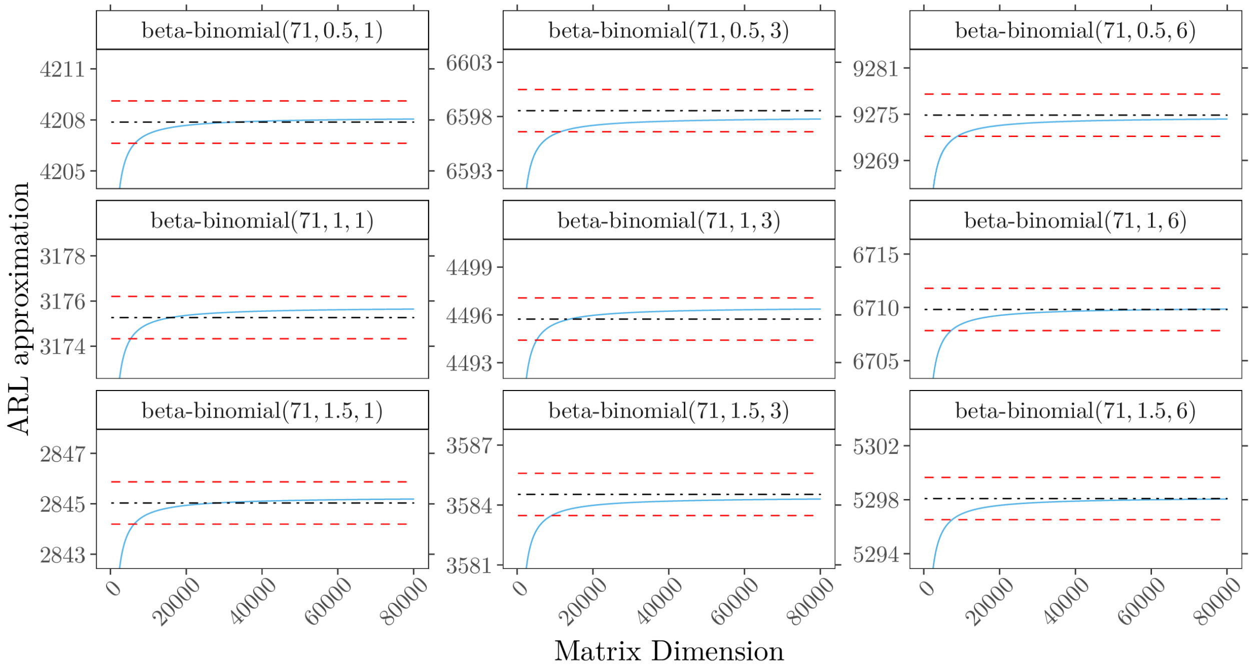

S. 5: In-control ARL approximation by Markov chain method (—) for detecting deterioration ($Q_A = 2$) and different beta-binomial(n, α, β) distributions with control limit $h^+ = 4.5$. Superimposed are Monte Carlo simulations with 10^8 replications (-·-) and three standard errors (- -).

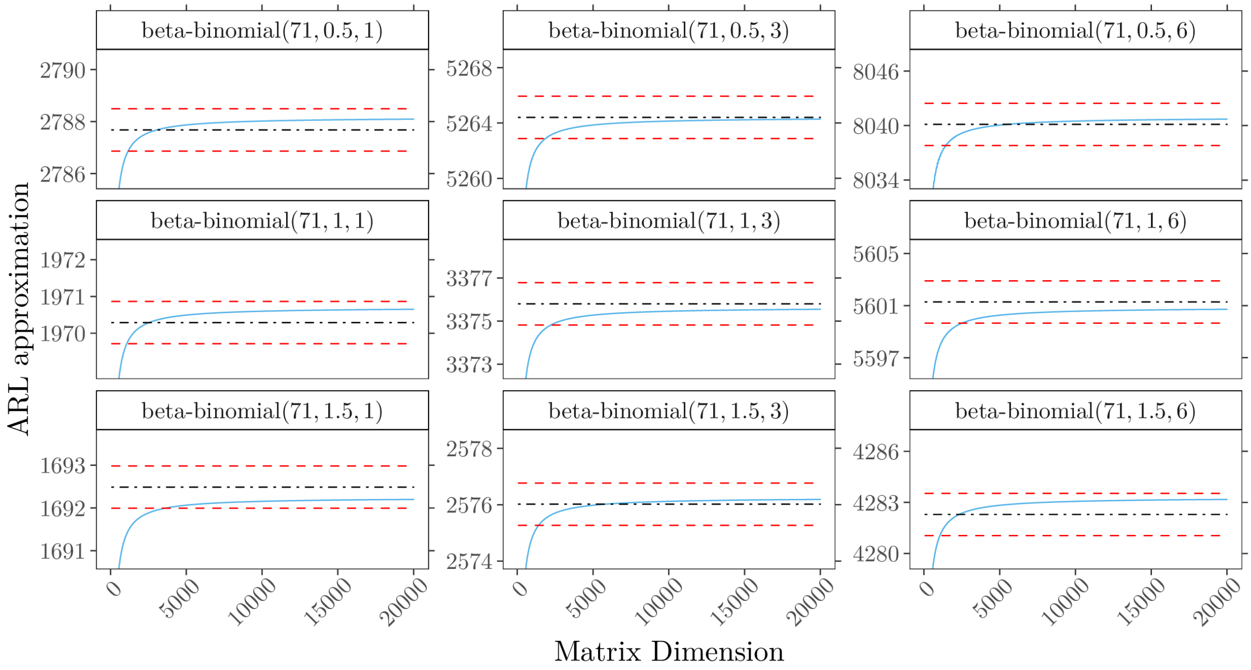

S. 6: In-control ARL approximation by Markov chain method (—) for detecting improvement ($Q_A = 1/2$) and different beta-binomial(n, α, β) distributions with control limit $h^- = 4$. Superimposed are Monte Carlo simulations with 10^8 replications (-·-) and three standard errors (- -).

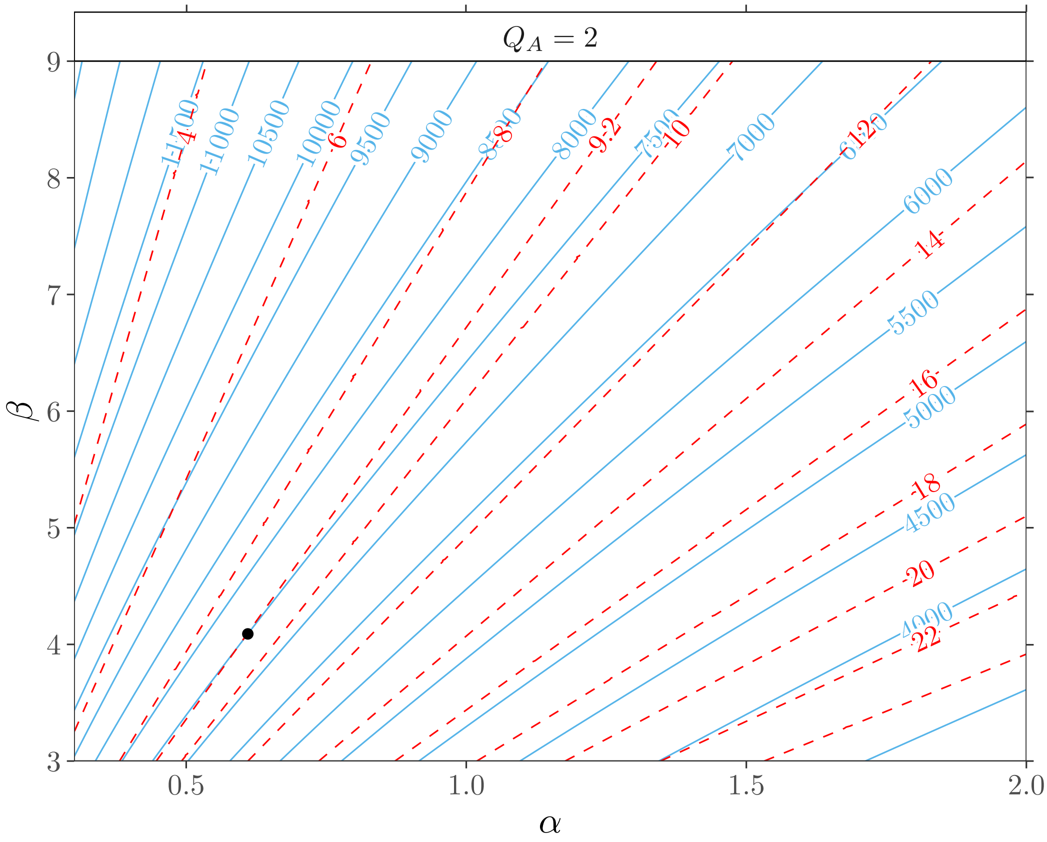

S. 7: In-control ARLs for discrete beta(α, β) models showing isolines of ARL (—) and expected risk score (- -). The control chart is calibrated for a discrete beta(0.61, 4.09) distribution (●) to an ARL_0 of 7 500.

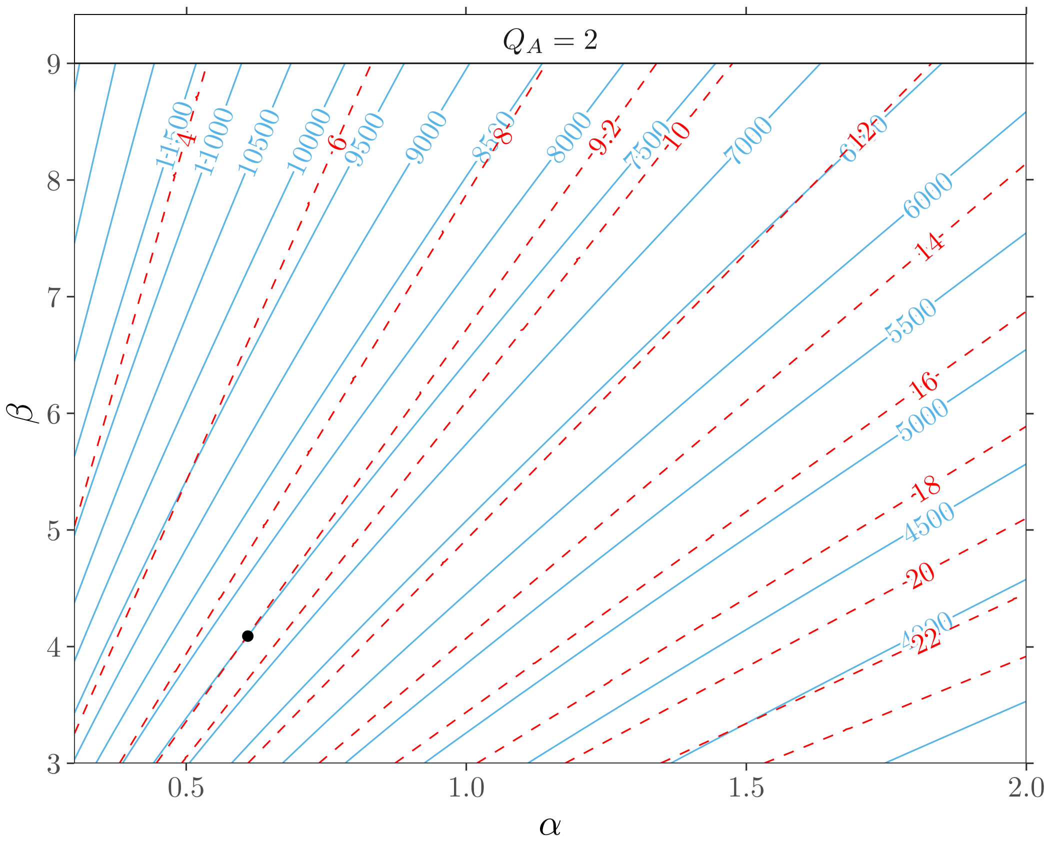

S. 8: In-control ARLs for beta(α, β) models showing isolines of ARL (—) and expected risk score (- -). The control chart is calibrated a for beta(0.61, 4.09) distribution (●) to an ARL_0 of 7 500.

T. 1: Patient distribution characteristics (average and median risk score) and their corresponding in-control ARLs.

Patient distribution	beta(α, β)			ARL_0 (discrete beta)		ARL_0 (beta)		Empirical data		
	Parameter	Average	Median	$Q_A = 2$	$Q_A = 1/2$	$Q_A = 2$	$Q_A = 1/2$	Average	Median	Cases
Artificial high-risk	(1.50, 4.00)	19.4	17.3	4 375.0	4 029.5	4 407.9	4 061.2	–	–	–
Surgeon #2	(0.92, 4.18)	12.8	9.8	6 047.0	5 880.2	6 063.5	5 896.9	12.4	10	287
Surgeon #1	(0.66, 3.41)	11.5	7.7	6 487.1	6 277.9	6 502.3	6 294.8	11.3	7	565
Surgeon #7	(0.85, 4.69)	10.9	8.0	6 787.2	6 756.1	6 792.2	6 760.3	10.5	8	260
Phase II	(0.79, 4.70)	10.2	7.2	7 078.3	7 085.5	7 079.3	7 085.4	9.8	7	4 776
Surgeon #3	(0.66, 4.05)	10.0	6.5	7 155.5	7 106.3	7 159.6	7 110.6	9.6	6	324
Complete data	(0.73, 4.49)	9.9	6.8	7 193.5	7 196.0	7 194.3	7 195.9	9.5	7	6 994
**Phase I***	**(0.61, 4.09)**	**9.2**	**5.7**	**7 500.3**	**7 500.3**	**7 500.4**	**7 500.2**	**8.9**	**6**	**2 218**
Surgeon #6	(0.62, 6.78)	6.0	3.5	9 701.4	10 240.5	9 652.3	10 180.0	5.6	3	474
Surgeon #5	(0.59, 8.09)	4.8	2.7	10 680.9	11 430.6	10 610.9	11 343.9	4.4	3	308
Artificial low-risk	(0.30, 8.00)	2.6	0.7	12 755.6	13 854.2	12 727.2	13 798.4	–	–	–
Single year 1994	(0.70, 3.84)	11.0	7.4	6 720.9	6 593.8	6 731.1	6 604.9	10.6	7	969
Single year 1995	(0.70, 4.16)	10.2	6.9	7 039.7	6 988.2	7 044.4	6 992.9	9.9	7	1 134
Single year 1996	(0.83, 4.52)	11.0	8.0	6 727,7	6 673.7	6 734.4	6 679.8	10.7	8	877
Single year 1997	(0.97, 6.04)	9.8	7.4	7 349.4	7 492.2	7 340.5	7 479.2	9.4	7	950
Single year 1998	(0.91, 6.54)	8.7	6.3	9 292.3	9 838.7	9 236.9	9 770.1	8.3	6	846

* Reference scenario

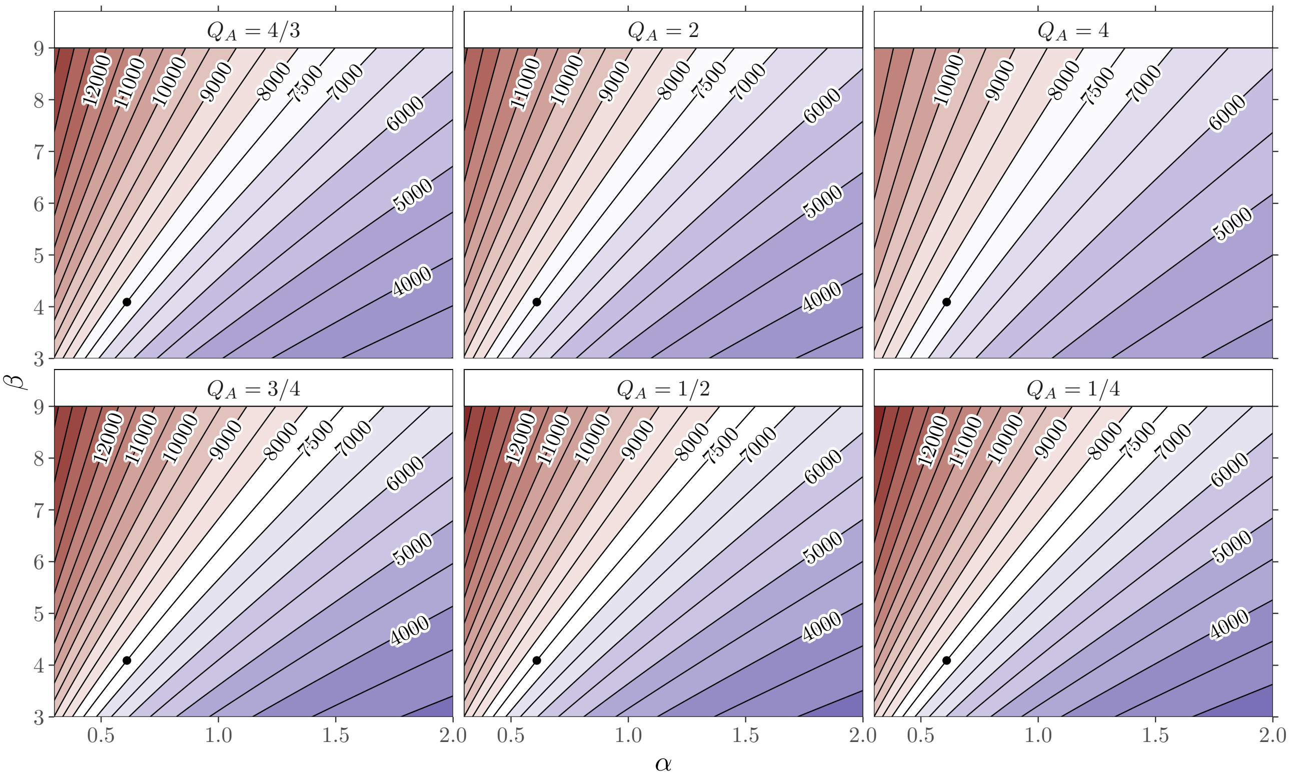

S. 9: In-control ARLs for discrete beta(α, β) models showing isolines of ARLs (—) for different out-of-control control shift sizes Q_A. All six charts are calibrated for a discrete beta(0.61, 4.09) distribution (●) to an ARL_0 of 7 500.

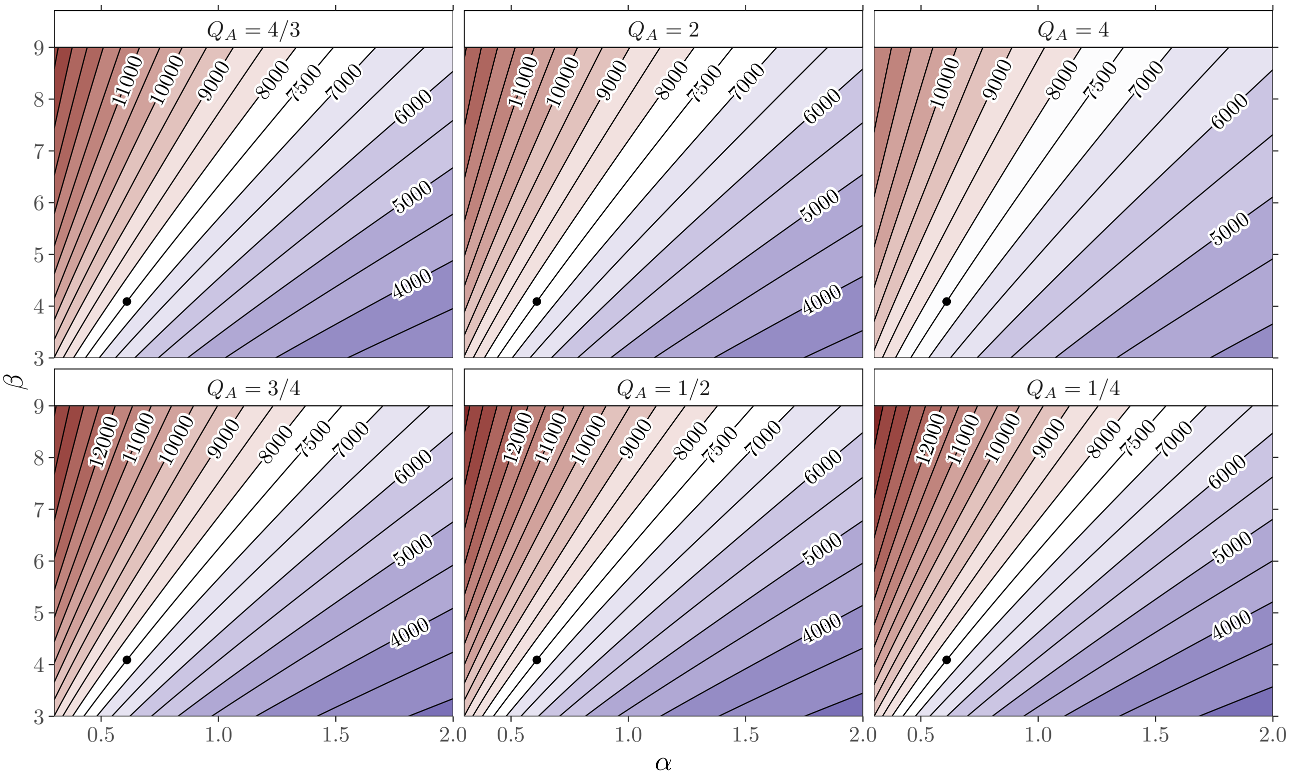

S. 10: In-control ARLs for beta(α, β) models showing isolines of ARLs (—) for different out-of-control control shift sizes Q_A. All six charts are calibrated for a beta(0.61, 4.09) distribution (●) to an ARL_0 of 7 500.
